# Integrative Metabolomic and Transcriptomic Analysis for the Study of Bladder Cancer

**DOI:** 10.3390/cancers11050686

**Published:** 2019-05-16

**Authors:** Alba Loras, Cristian Suárez-Cabrera, M. Carmen Martínez-Bisbal, Guillermo Quintás, Jesús M. Paramio, Ramón Martínez-Máñez, Salvador Gil, José Luis Ruiz-Cerdá

**Affiliations:** 1 Unidad Mixta de Investigación en Nanomedicina y Sensores, Universitat Politècnica de València-Instituto de Investigación Sanitaria La Fe, 46026 Valencia, Spain; lomonal@alumni.uv.es (A.L.); rmaez@qim.upv.es (R.M.-M.); jose.l.ruiz@uv.es (J.L.R.-C.); 2 Grupo de Oncología Celular y Molecular, Hospital Universitario 12 de Octubre, 28041 Madrid, Spain; jesusm.paramio@ciemat.es; 3 Unidad de Oncología Molecular, Centro de Investigaciones Energéticas, Medioambientales y Tecnológicas (CIEMAT) (ed70A), 28040 Madrid, Spain; 4 Instituto Interuniversitario de Investigación de Reconocimiento Molecular y Desarrollo Tecnológico, Universitat Politècnica de València, Universitat de València, 46022 Valencia, Spain; salvador.gil@uv.es; 5 CIBER de Bioingeniería, Biomateriales y Nanomedicina (CIBER-BBN), 28029 Madrid, Spain; 6 Departamento de Química Física, Facultad de Químicas, Universitat de València, 46100 Burjassot, Spain; 7 Analytical Unit, IIS La Fe, 46026 Valencia, Spain; gquintas@leitat.org; 8 Health & Biomedicine, Leitat Technological Center, 08225 Terrassa, Spain; 9 Centro de Investigación Biomédica en Red de Cáncer (CIBER-ONC), 28029 Madrid, Spain; 10 Unidad Mixta UPV-CIPF de Investigación en Mecanismos de Enfermedades y Nanomedicina, Universitat Politècnica de València, Centro de Investigación Príncipe Felipe, 46012 Valencia, Spain; 11 Departamento de Química, Universitat Politècnica de València, 46022 Valencia, Spain; 12 Departamento de Química Orgánica, Facultad de Químicas, Universitat de València, 46100 Burjassot, Spain; 13 Servicio de Urología, Hospital Universitario y Politécnico La Fe, 46026 Valencia, Spain

**Keywords:** cancer biomarkers, bladder cancer, metabolomics, transcriptomics, metabolic pathways, tumor metabolome, cancer metabolic reprogramming

## Abstract

Metabolism reprogramming is considered a hallmark of cancer. The study of bladder cancer (BC) metabolism could be the key to developing new strategies for diagnosis and therapy. This work aimed to identify tissue and urinary metabolic signatures as biomarkers of BC and get further insight into BC tumor biology through the study of gene-metabolite networks and the integration of metabolomics and transcriptomics data. BC and control tissue samples (n = 44) from the same patients were analyzed by High-Resolution Magic Angle Spinning Nuclear Magnetic Resonance and microarrays techniques. Besides, urinary profiling study (n = 35) was performed in the same patients to identify a metabolomic profile, linked with BC tissue hallmarks, as a potential non-invasive approach for BC diagnosis. The metabolic profile allowed for the classification of BC tissue samples with a sensitivity and specificity of 100%. The most discriminant metabolites for BC tissue samples reflected alterations in amino acids, glutathione, and taurine metabolic pathways. Transcriptomic data supported metabolomic results and revealed a predominant downregulation of metabolic genes belonging to phosphorylative oxidation, tricarboxylic acid cycle, and amino acid metabolism. The urinary profiling study showed a relation with taurine and other amino acids perturbed pathways observed in BC tissue samples, and classified BC from non-tumor urine samples with good sensitivities (91%) and specificities (77%). This urinary profile could be used as a non-invasive tool for BC diagnosis and follow-up.

## 1. Introduction

BC is an unsolved clinical, social and economic problem [1]. At diagnosis, two principal classes of BC are distinguished: non-muscle invasive BC (NMIBC), which includes carcinoma in situ (CIS), Ta, and T1 tumors; and muscle-invasive BC (MIBC), which encompasses tumors with a stage ≥T2. NMIBC represents around 80% of BC cases and displays, in general, a better clinical outcome (5-year survival ~90%) than the MIBC (5-year survival <50%) [2,3]. However, NMIBC presents with one of the highest recurrence rates among all cancers, as 50–70% of cases diagnosed recur during the first two years after diagnosis and 10–15% progress to invasive within 5 years [2]. Consequently, NMIBC patients require a lifetime follow-up by cystoscopy. NMIBC are treated by transurethral resection (TUR) followed by intravesical instillations of chemotherapy or immunotherapy (Bacillus Calmette-Guerin, BCG). By contrast, MIBC patients require radical cystectomy usually followed by cisplatin-based chemotherapy. Unfit patients for this treatment have few therapeutic options but modern immunotherapy has changed the paradigm of advanced or metastatic BC. Currently, five drugs have been approved by the Food and Drug Administration Agency (FDA, White Oak, MD, USA) for the second line therapy and/or first line for cisplatin-ineligible patients, showing hopeful results [4].

Appropriated diagnostic tools are needed to correctly handle BC patients. Nowadays, cystoscopy and urinary cytology are the gold standards for BC diagnosis and follow-up [5]. However, urinary cytology has low sensitivity in low grade tumors detection and cystoscopy is invasive, expensive, operator-dependent [5], and has limitations in the differentiation between inflammation and malignancy and in the diagnosis of CIS, which is considered tumors at high risk of progression [6]. In this scenario, the development of non-invasive strategies for the improvement of early diagnosis and detection of recurrences in the follow-up is of importance. Among biofluids and tissue samples to develop these strategies, the search for BC biomarkers in urine is a logical approach because urine comes into contact with the bladder, which sheds cells and metabolites into this biofluid [7]. Several assays using urine have received the FDA approval for the BC diagnoses or follow-up. Nonetheless, none of them outperforms cystoscopy in the everyday clinical practice [8].

Remarkably, whereas the current therapies are selected according to pathological criteria, recent analyses of BC at a molecular level have provided a more complex panorama, defined by multiple molecular subtypes characterized by specific mutational and transcriptional programs [9,10]. One emerging hallmark of cancer is the metabolic reprogramming to supply bioenergetic and biosynthetic demands of continuous cell proliferation, including the high uptake of glucose and its use through glycolysis along with increased lipid, nucleotide and amino acid (AA) biosynthesis [11]. Although metabolic reprogramming may occur by alterations in oncogenes and tumor suppressor genes, the altered metabolism can also play a primary role in oncogenesis [12]. Mutations in genes coding for key metabolic enzymes lead to an altered control of genomic and epigenomic processes in tumor cells [13]. Chromatin-modifying enzymes utilize metabolites and co-factors from intermediate metabolism, and accordingly, their availability is crucial to remodel the chromatin structure and to modulate gene expression in cancer cells [12]. Bearing all this in mind, integrative transcriptomic and metabolomic studies show high potential in biomarker research for the implementation of precision medicine. In this line, technological developments in Mass Spectrometry (MS) and Nuclear Magnetic Resonance spectroscopy (NMR) have fostered the study of cancer metabolism. Several metabolomic studies have been performed in tissue samples or biofluids, such as urine or serum to identify BC biomarkers for diagnosis or monitoring of patients [14,15]. Metabolites such as taurine, carnitine and cholinergic compounds [14,15,16] have been proposed as BC biomarkers in urine. However, further research and validation is required in order to demonstrate their clinical usefulness. Additionally, although multiple genomic analyses have been carried out in BC showing altered expression or mutations in metabolic enzymes, there are no reported integrative transcriptomic and metabolomic studies in BC tissue samples or liquid biopsies, as far as we know.

Under the hypothesis that urinary metabolome reflects the alterations produced at different levels of molecular regulation in bladder tumors, we report an integrated metabolomic and transcriptomic study aimed to determine the association between changes in metabolism and gene expression in BC samples. BC and non-tumor bladder tissue and urine samples from the same patients were analyzed by NMR and provided a set of potential metabolomic hallmarks, which were supported by transcriptomic data. NMR and chemometric analysis provided metabolic signatures that classified BC tissue and urine samples with respect to control samples with high sensitivity and specificity. Moreover, the metabolites in BC urine signatures agreed with the alterations found in the transcriptomic and metabolomic tissue studies. This experimental design allowed for studying the relation between the tissue and the urinary metabolome in the same patients avoiding the intra-tumor heterogeneity associated to tumors.

## 2. Results

### 2.1. Tissue NMR Profile

**Figure 1:**
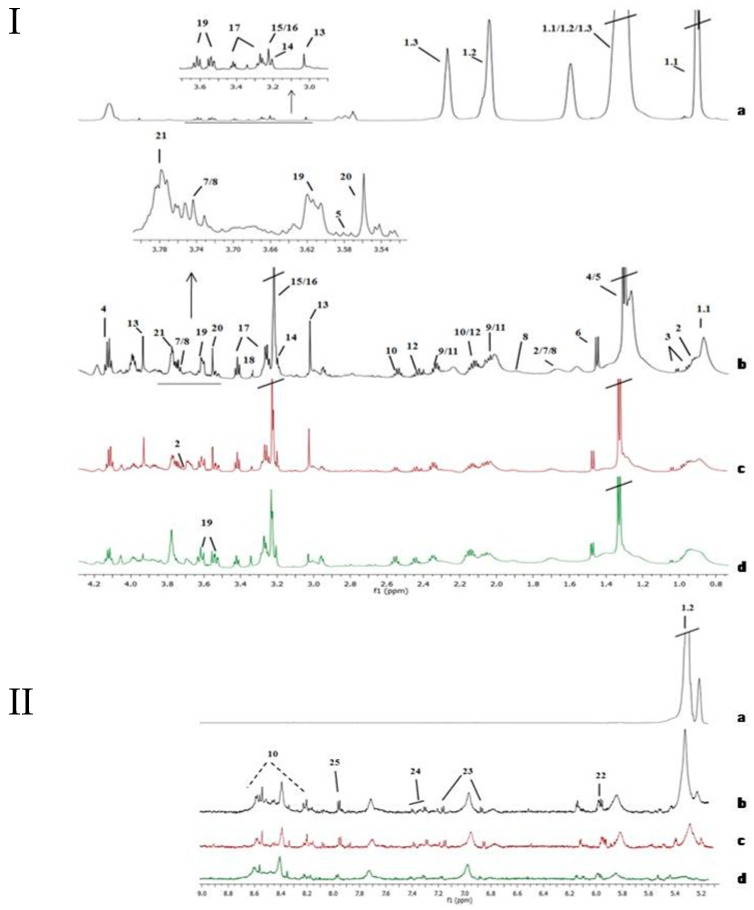
Assignment of the main signals in ^1^H NMR spectra of tissue samples (aliphatic region, I, and aromatic region, II). Representative NMR spectra of control non-affected tissue (I and II-**a**), and various bladder cancer pathologic stages Ta/T1 (I and II-**c** and I and II-**d**) and T2 (I and II-**b**). Vertical scale of all the spectra was kept constant and spectral region of water was removed from the figure. The intensity of peaks in the chemical shift region 5.0—9.0 ppm was scaled equally in all the spectra to show the low abundant metabolites. Metabolic identification: 1.1.Lipid fragment –(n)CH_2_-CH_2_-CH_2_-CH_3_ (I-**a**), 1.2.Lipid fragment –CH=CH-CH_2_-CH=CH-CH_2_-CH_2_-(n)CH_2_ (I and II-**a**), 1.3.Lipid fragment –(n)CH_2_-CH_2_-CH_2_-COOH (I-**a**), 2. Leucine, 3. Valine, 4. Lactate, 5. Threonine, 6. Alanine, 7. Lysine, 8. Arginine, 9. Glutamate, 10. Glutathione, 11. Proline, 12. Glutamine, 13. Creatine, 14. Choline, 15. PCholine, 16. GPCholine, 17. Taurine, 18. Methanol, 19. Myo-inositol, 20. Glycine, 21. Glycerol, 22. UDP-sugars, 23. Tyrosine, 24. Phenylalanine, 25. Cytidine diphosphate (CDP).

High-Resolution Magic Angle Spinning (HRMAS) NMR spectra for tissue samples showed a good signal to noise ratio. Figure 1 displays representative tissue spectra from non-tumor sample and three different stages of BC (Ta, T1 and T2). Non-tumor tissue samples spectra were dominated by intense lipid signals in the aliphatic region that were practically absent in tumors. Tumors showed signals of small metabolites as glutathione (GSH), tyrosine and cytidine diphosphate (CDP), among others. Complete assignment of signals in bladder tissue NMR spectra are shown in Supplementary Table S1.

### 2.2. Mean Comparison of Metabolites Detected in Tissue

**Figure 2:**
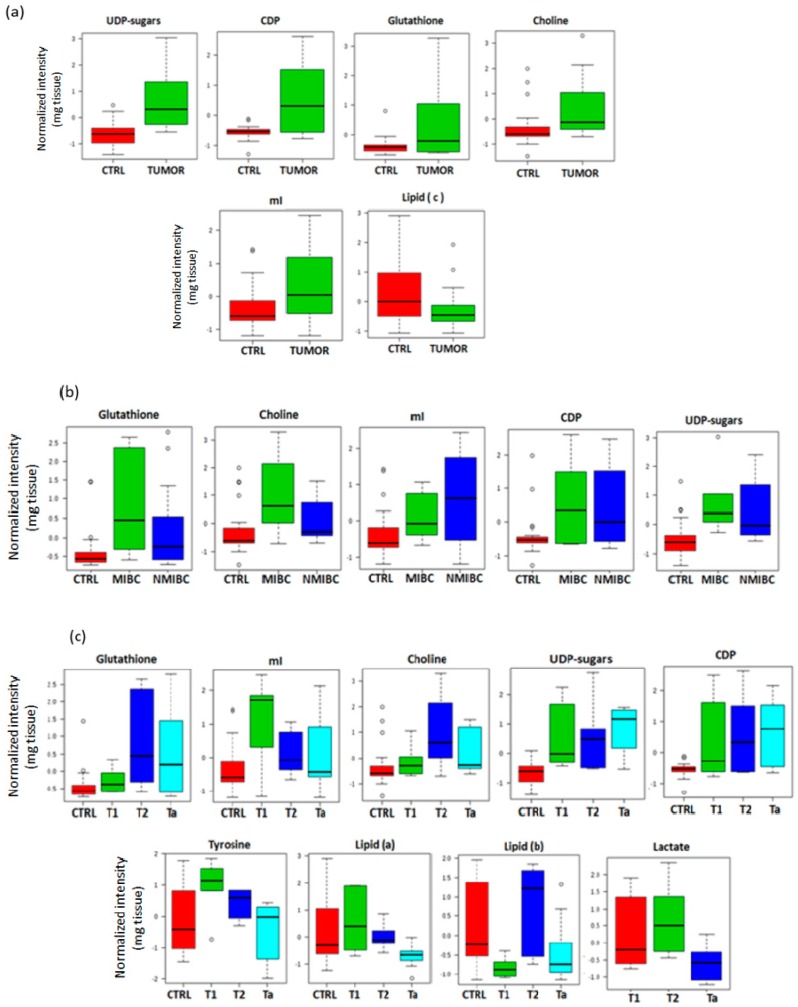
Box and whisker plots comparing the mean of metabolites detected in tissue. The plots illustrate the discrimination between: (**a**), tumor and non-tumor tissue samples; (**b**), controls, non-muscle invasive bladder cancer (NMIBC) and muscle-invasive bladder cancer (MIBC); (**c**), different stages of BC and control tissue samples.

From a mean comparison of metabolites intensities from HRMAS in tumor and control samples a higher intensity of choline (Cho), CDP, myo-inositol (mI), UDP-sugars and GSH was found in tumors tissue samples. Intensity for lipid fragment –(n)CH_2_-CH_2_-CH_2_-COOH (named lipid (c) as in [17]) was higher in control samples (Figure 2a; Supplementary Table S2). Other metabolites such as glutamate or glutamine did not show significant differences between tumor tissue and control samples (*p* > 0.05). Most of the metabolites showing significant differences between tumor and control tissue samples were also elevated in both tumor groups (NMIBC and MIBC) compared to control samples (Figure 2b; Supplementary Table S2).

Considering the differences among the diverse tumors stages and controls, lipid fragments –CH_2_-CH_2_-CH_2_-CH_3_ (lipid (a) as in [17]) and –CH=CH-CH_2_-CH=CH-CH_2_-CH_2_-(n)CH_2_- (lipid (b) as in [17]), tyrosine, Cho, CDP, mI, UDP-sugars and GSH showed significant changes between all groups. Specifically, T2 tumors (the most aggressive here considered) presented the highest intensity of Cho and GSH. T2 and T1 stages showed elevated mI and tyrosine. On the other hand, Ta tumors (the least aggressive tumors) showed a significant increment of UDP-sugars and CDP (Figure 2c; Supplementary Table S2). Lactate signals at 1.33 ppm and 4.1 ppm were overlapped with lipid signals in controls. The overlap hinders the discrimination between lactate and lipid signals in control samples. Therefore, the lactate intensity was compared only between the different tumor stages and not respect to the control group. Higher intensity of lactate in T2 than in T1 or Ta was observed (Figure 2c). 

### 2.3. PLS-DA Analysis in NMR Tissue Data

A chemometric analysis was performed on 1D HRMAS tissue spectra including the original set of variables, and after a feature selection based on the variable importance in projection (VIP > 1). Cross Validation (CV) assessed the predictive performance of the initial Partial Least-Squares Discriminant Analysis (PLS-DA) model (n = 34, latent variables (LV = 2)) providing a sensitivity of 82.4% and a specificity of 88.2% (see confidence intervals in Supplementary Table S3). Permutation test provided a *p* < 0.05. The analysis of the samples included in the external validation set presented sensitivity and selectivity values of 100% for the two PLS-DA models (original set of variables and VIP > 1) (Supplementary Figure S2). 

**Figure 3:**
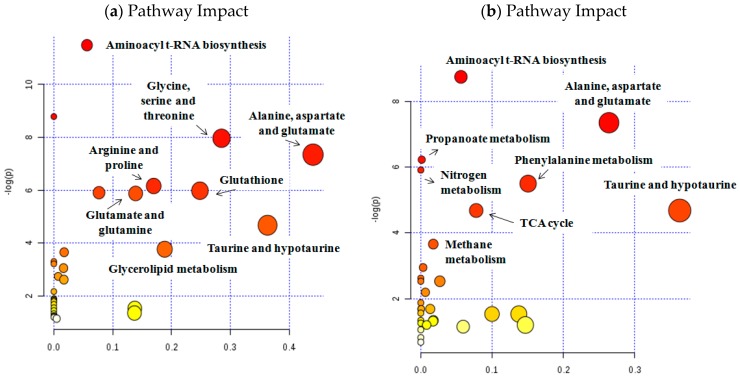
Analysis of altered metabolic pathways in bladder tissue (**a**) and urine (**b**) samples. In both cases (tissue and urine samples), the metabolites identified among the set of metabolic features included in Partial Least-Squares Discriminant Analysis (PLS-DA) model (variable importance in projection (VIP) > 1) have been used.

A VIP threshold of 1 was selected as the compromise between the model complexity (i.e., number of retained variables) and the predictive performance of the PLS-DA model (Supplementary Figure S3). The most discriminant metabolites between tumor and non-tumor tissue samples identified in this PLS-DA model included: lipids (a, b and c fragments), threonine, lactate, alanine, glutamate, proline, glutamine, GSH, creatine, Cho, phosphocholine (PCho), glycerophosphocholine (GPCho), taurine, methanol, mI, glycine, glycerol, UDP-sugars, tyrosine and CDP. This set of metabolites reflected alterations in metabolic pathways related with the metabolism of several AA pathways and also with GSH, taurine and hypotaurine or glycerolipids (Table 1; Figure 3).

### 2.4. Transcriptomic Analysis in Tissue Samples

**Figure 4:**
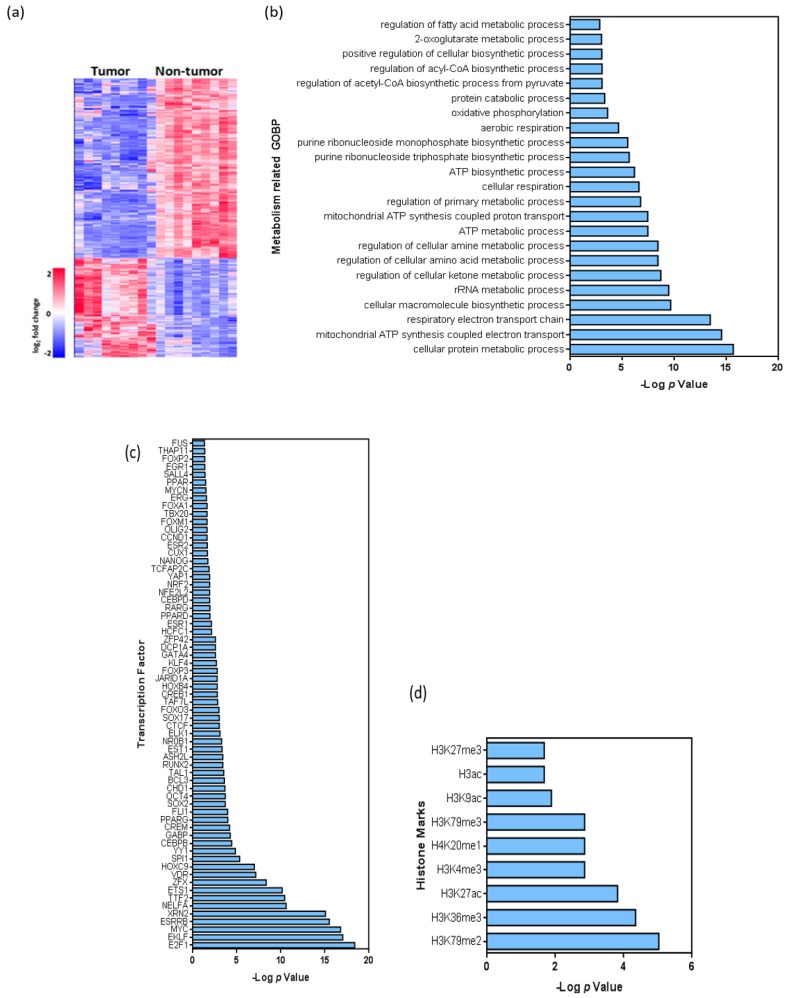
Transcriptome analysis of non-tumor and tumor samples of BC patients. (**a**), Heatmap showing the hierarchical clustering of tumor and non-tumor samples, as well as the number of downregulated and upregulated transcripts. (**b**), Summary of Gene Ontology analysis: main metabolism-related biological processes altered in tumor samples are shown. (**c**,**d**) Summary of putative binding motif enrichment analysis using the Enrich webtool [18], showing the relative relevance of various c, transcription factors d, histone marks associated with the differentially expressed transcripts. In (**b**, **c** and **d**), values are shown as –log (*p*).

Principal Component Analysis (PCA) and heatmap analyses were performed to search transcriptomic differences between BC tissue and non-tumor tissue samples. PCA showed clear differences between tumor and non-tumor samples on the basis of the whole transcriptome (Supplementary Figure S4). Following a fold change of at least 2 or −2 and a false discovery rate of 0.05 as selection criteria, a total of 4409 transcripts differentiated tumor and non-tumor samples (Figure 4a), being predominantly downregulated in tumors (3112 transcripts). The Gene Ontology of Biological Processes (GOBP) analysis revealed that different metabolism-related genes were significantly downregulated in tumors (Figure 4b), including various mitochondrial RNAs and genes related with the regulation of AA and amine metabolism, purine biosynthetic processes and oxidative phosphorylation (OXPHOS). On the contrary, among the upregulated transcripts in tumors, no significant metabolism-related categories were found in our study.

A total of 364 unique genes were identified considering only protein coding genes linked to metabolic processes from GOBP and GeneCards databases (Supplementary Table S4), from which 20 genes were overregulated. Interestingly, some of the overregulated genes have already been linked to cancer such as: the peroxisome proliferator activated receptor gamma (PPARG) involved in lipid biogenesis regulation [12]; the hexokinase 2 (HK2) and the solute carrier Family 2 Member 1 (SLC2A1) [19], both them related with glycolysis; the ribose-5-phosphate isomerase A (RPIA) linked to pentose phosphate pathway; and genes belonging to cytochrome P450 [20] (CYP450) (CYP2J2, CYP2C9, CYP4F11). On the other hand, key metabolic genes were found downregulated in BC tissue samples, including genes related to: (1) pyruvate metabolism: pyruvate dehydrogenases (PDHA1, PDHB, PDHX) and pyruvate dehydrogenase kinase 4 (PDK4); (2) TCA cycle: FH, IDH3A, IDH3B, MDH1, MDH2, ACO1, OGDH and SUCLG1; (3) polyamine metabolism: AMD1, SMS, ODC1, SAT2, AOC3; (4) AA metabolism: GLS, GOT2, MUT, ASS1, MAOA, MAOB; (5) redox status: peroxidases, catalase, superoxide dismutase, and glutaredoxin. Importantly, although our series is enriched in NMIBC, the expression of the upregulated and downregulated metabolism-related genes also demonstrated similar behavior in multiple tumor samples present in the Cancer Genome Atlas (TCGA) cohort (enriched in MIBC) without discrimination of stage, grade of previously identified mRNA tumor subtypes (Supplementary Figure S5).

In silico analysis revealed that multiple genes were bound by transcription factors currently known as potentially involved in BC pathogenesis (Supplementary Table S5). Remarkably, these could also act as transcriptional repressors, such as ETS1, TTF2, E2F1, YY1, FLI1, ASH2L, E2F4, VDR, GABP, JARID1A, CTCF, KLF4 and CHD1 (Figure 4c). Besides, some of these factors are known to modulate gene expression through direct chromatin remodeling, such as GABP, JARID1A, CTCF and CHD1. Accordingly, we used the Encode Histone Roadmap [20] to determine whether the downregulated metabolism-related genes were specifically associated with particular histone marks. This analysis revealed that active transcription histone marks such as H3K79me2, H3K79me3, or H3K27ac, as well as repressive marks such as H3K36me3, H4K20me1 orH3K9me3, were present in the downregulated metabolic genes (Figure 4d; Supplementary Table S6). On the other hand, the transcriptome analysis revealed the presence of specific splicing variants of some metabolic genes (GLS, MAOA, ADH1C, ADH5, OGDH, SUCLG1, MDH1, MDH2, SMS, ODC1, FARSB, ACADM, PPM1L, PDK4, PDHX, PDHA1, GOT2, ASS1, RARS, OAT, AOC3, HIBADH, DLD, FH, AUH, MUT) in tumors compared to non-tumor samples (Supplementary Figure S6).

**Figure 5:**
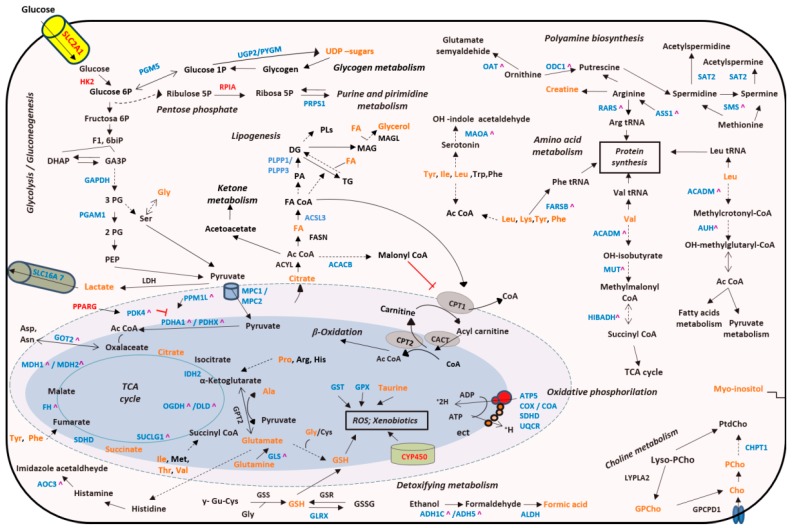
Integrated altered metabolic pathways in bladder tumors according to our metabolomic and transcriptomic data. Note: Enzymes shown in red indicate significant overexpression in BC tissue; enzymes shown in blue indicate significant downexpression; enzymes shown in black indicate no significant changes in our studies; metabolites shown in orange indicate that they were identified in our studies; ^ Indicate that these genes presented a differential alternative splicing in our tumor tissue samples; Dashed lines indicate that the reaction is not direct. Ac CoA: acetyl coenzyme A; ADP: adenosine diphosphate; Ala: alanine; Arg: arginine; Asn: asparagine; Asp: aspartate; ATP: adenosine triphosphate; 1;3 biPG: 1,3-bisphosphoglycerate; Cho: choline; CoA: coenzyme A; DG: diacylglycerides; DHAP: dihydroxyacetone phosphate; FA: fatty acids; F1,6biP: fructose-1,6-bisphosphate; GA3P: glyceraldehyde 3-phosphate; Glu: glutamate; Gly: glycine; GPCho: glycerophosphocholine; GSH: glutathione; His: histidine; Ile: isoleucine; Leu: leucine; Lys: lysine; MAG: monoacylglycerols; NO: nitric oxide; PA: phosphatidic acid; PEP: phosphoenolpyruvate; 2PG: 2-bisphosphoglycerate; 3PG: 3-bisphosphoglycerate; Phe: phenylalanine; PCho: phosphocholine; PLs: phospholipids; Pro: proline; ROS: reactive oxygen species; Ser: serine; TCA cycle: tricarboxylic acid cycle; TG: triacylglycerides; Trp: tryptophan; Tyr: tyrosine; UDP-sugars: uridine diphosphate-sugars; tRNA: transfer ribonucleic acid. The names of the significant metabolic genes are detailed in the Supplementary Table S4.

Finally, the integrative analysis using metabolomics and transcriptomics data revealed deregulated activity of multiple biochemical pathways such as: TCA cycle, OXPHOS and polyamine and AA metabolism, among others (Figure 5).

### 2.5. Metabolomic Analysis in Urine Samples

**Figure 6:**
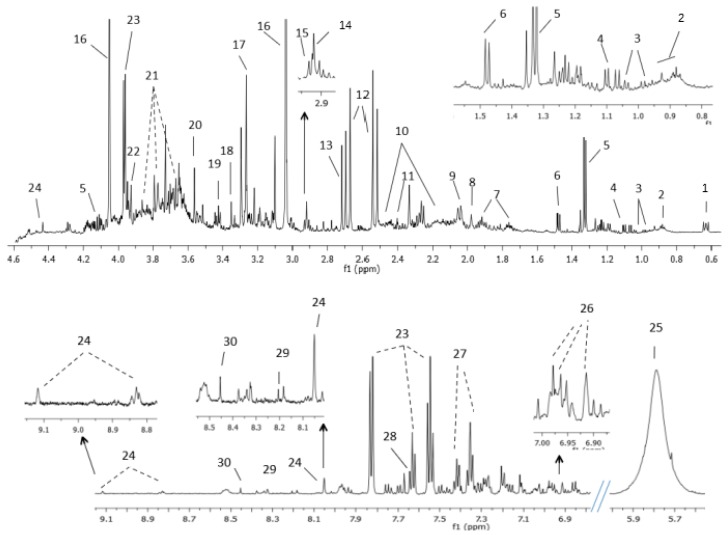
Assignment of the main signals in ^1^H NMR spectra of BC urine. Representative spectra of urine from patient with BC. The vertical scale of all the spectra was kept constant and spectral region containing spinning side bands (6–6.8 ppm) and water were removed from the figure. The intensity of peaks in the chemical shift region 5.5–9.1 ppm was scaled equally in all the spectra to show the low abundant metabolites. Metabolic identification: 1.DSS, 2.Branched-chain amino acids, 3.Valine, 4.Methylsuccinic acid, 5.Lactate, 6.Alanine, 7.Lysine, 8.Acetic acid, 9.N-acetylneuraminic acid, 10.Glutamine, 11.Succinate, 12.Citrate, 13.Dimethylamine, 14.Trimethylamine, 15.Dimethylglycine, 16.Creatinine, 17.Trimethylamine N-oxide, 18.Methanol, 19.Taurine, 20.Glycine, 21.Sucrose, 22.Creatine, 23.Hippuric acid, 24.Trigonelline, 25.Urea, 26.3-(3-hydroxyphenyl)-3-hydroxypropionic acid (HPHPA), 27.Phenylalanine, 28.Pseudouridine, 29.Hypoxanthine, 30.Formic acid.

NMR urine spectra showed a good signal to noise ratio. The assignment of metabolites identified in BC urine samples and controls are in Figure 6 and in Supplementary Table S7. In all samples low-molecular-weight metabolites like AAs or organic acids were observed. The resonances of urea, creatinine, trimethylamine-N-oxide, hippuric acid and citrate were the most predominant.

### 2.6. PLS-DA Analysis in NMR Urine Data

A PLS-DA analysis was performed with urine NMR data to classify BC and control urine samples. The PLS-DA model (n = 35, LV = 5) provided a sensitivity of 90.9%, specificity of 76.9%, a negative predictive value (NPV) of 83.0% and a positive predictive value (PPV) of 86.9% and an area under receiver operating characteristic (AUROC) = 0.9 (Supplementary Figures S7 and S8). Permutation test provided a p < 0.05 and assessed the statistical significance of the CV-predictive performance of the model.

The most discriminant metabolites identified in urine (VIP > 1) included: valine, methylsuccinic acid, lactate, alanine, lysine, N-acetylneuraminic acid, glutamine, succinic acid, citrate, creatinine, trimethylamine-N-oxide, methanol, taurine, sucrose, hippuric acid, phenylalanine, pseudouridine and trigonelline. These metabolites were linked with the following disturbed metabolic pathways: alanine, aspartate and glutamate metabolism, taurine and hypotaurine, TCA cycle and aminoacyl t-RNA biosynthesis (Figure 3; Table 1).

## 3. Discussion

Cancer cells undergo genetic reprogramming of their metabolism in order to fulfill the increased energetic and biosynthetic demands for cell proliferation. Therefore, better understanding of altered genetic basis of the metabolic pathways and some altered metabolites could be useful to find non-invasive diagnostic, prognostic and surveillance biomarkers but also to develop new therapy strategies in the context of BC. With this aim, a comprehensive study focused on knowing better the relationship between the metabolome and transcriptome of BC, and its link with urinary metabolome has been here carried out. 

Integrated metabolomic and transcriptomic data showed perturbations in several tissue metabolic pathways, reflecting how the activity of metabolic genes and enzymes would be regulating the demands of BC. Moreover, the upregulated and downregulated metabolism-related genes found in our series enriched in NMIBC demonstrated similar behavior in multiple tumor samples present in the TCGA cohort (enriched in MIBC), indicating that the deregulation of these genes is probably a common feature of BC pathogenesis.

According to our data from PLS-DA model, the metabolism of AAs would be important for BC cells. Among other AAs, the signals of glutamate, glutamine and tyrosine were found important in our statistical model to classify BC. Moreover, according to the mean comparison high levels of tyrosine were observed in T1 and T2 tumors compared to Ta or control tissue samples. Nevertheless, the differences of means of glutamine and glutamate for control and BC tissue samples did not reach statistical significance in the univariate analysis (U-Mann–Whitney test). These findings, somewhat controversial, could be explained taking into account, among other factors, that independent variables may complement each other in the prediction of the dependent variable [21]. Complementary predictors could be found that, separately, do not explain the differences between classes, but together they do it [21]. Thus, in our study the means of glutamine and glutamate are not significantly different between BC and control tissue, but they are important (together with other metabolites) to discriminate between these classes in the PLS-DA model. Additionally, we found the glutaminase (*GLS*) gene downregulated, which could be in concordance with the findings on glutamine and glutamate. GLS converts glutamine to glutamate, which is used by alanine aminotransferase (GPT2) to produce α-ketoglutarate. Although previous studies have reported a very active GLS in cancer cells, a post-transcriptional regulation mediated by miRNAs could play an important role, so more studies are necessary in this field, to better understand the role of glutamine and glutamate metabolism in BC. Our data would suggest that the more invasive tumors would consume tyrosine and glutamine for energy generation, as a source of reducing power, as a donor of carbon and nitrogen for generating nucleotides and other AAs, and also to maintain the pool of intermediate metabolites such as acetyl CoA and α-ketoglutarate, both of them important for the anaplerotic reactions of TCA cycle. However, we found a strong downregulation in all TCA cycle genes. These findings are in concordance with previous studies in which frequently mutated or deregulated TCA cycle genes are described in human tumors [22]. 

Moreover, our data support a strong downregulation of OXPHOS process and the overexpression of some glucose metabolic genes (SLC2A1, HK2 and RPIA) linked to high levels of lactate, mostly in T2 BC. These data agree with the Warburg effect, which describes that tumor cells prefer to oxidize glucose to obtain energy and precursor molecules through anaerobic respiration (lactic fermentation) even in oxidative conditions, rather than obtaining ATP through a more efficient process in the mitochondria (OXPHOS) [23]. On the other hand, they are in concordance with previous studies about BC, which have correlated high expression of SLC2A1 with disease progression and poor survival [24] and high levels of HK2 with an acceleration in glucose flux through glycolysis towards pyruvate [25], and lactic fermentation. The high levels of lactate in aggressive tumors were also observed in previous studies where the progression of BC from a less to a highly invasive stage was associated with increased production of lactate [26], and agree with the downexpression found in pyruvate dehydrogenase genes. However, they are in discordance with the downregulation of PDK4, so more studies in this field are necessary to know better the regulation of these metabolic pathways in BC.

The nexus between glucose and fatty acid metabolism is carried out by the transcriptional factor PPARG. PPARG regulates PDK4 and is involved in the regulation of adipogenesis, cell proliferation, angiogenesis and immune surveillance [27]. We found an overexpression of PPARG, as occurs in other tumors (colon, lung, prostate) [12,28] and elevated levels of lipid (b) in T2 tumors and lipid (c) in control samples. Glycerol also was identified as important in our statistical model. These data would suggest that lipid metabolism is important for BC cells [16,29], which could use fatty acids to obtain energy (β-oxidation), for the production of signaling molecules but also for the biosynthesis of phospholipids, the main lipids of cell membranes.

Regarding choline and inositol metabolism, the signals of Cho, PCho, GPCho, CDP and mI were relevant in our statistical model. Besides, significant high intensities of Cho, CDP and mI were found in BC tissue samples, specifically in invasive and T1 tumors. These data agree with previous studies, which present the abnormal cholinic phenotype as a hallmark of tumor development and progression [30]. Cho and PCho are precursors of phosphatidylcholine (PtdCho), one of the main constituents of the lipid bilayers of cell membranes together with phosphatidylethanolamine (PtdE). CDP also participates in PtdCho biosynthesis, at the same way that mI, which has been associated with PtdCho turnover and modulation of phospholipids. De novo synthesis of PtdCho and PtdE is referred as Kennedy pathway and it has been proposed as a chemotherapeutic target against cancer [31].

Polyamine metabolism is frequently deregulated in tumors [32] and is related to AA metabolism through arginine, methionine and ornithine. We observed a downregulation in polyamine enzymes, either biosynthetic (ODC1, AMD1, SMS) or catabolic (SAT2, AOC3); however, we have not identified any metabolite as discriminant related to this pathway. However, studies performed by MS in urine samples and BC cell lines have found higher levels of spermidine, arginine, methionine and ornithine in BC compared to control samples [15]. Polyamines can act in the regulation of gene expression through altering chromatin structure, signal transduction pathways and interactions between DNA-protein or protein-protein [33].

Maintaining the redox status is key for cancer cells. At low levels, reactive oxygen species (ROS) increase cell proliferation and survival but at high levels ROS cause DNA damage and trigger apoptosis [34]. In the context of BC, high levels of ROS have been linked with chemotherapy resistance and bladder tumorigenesis [35]. Therefore, the role of antioxidant molecules is essential. In our study BC tissue samples presented high levels of GSH (specifically MIBC), but a downregulated expression of other antioxidant enzymes involved in ROS detoxification. On the other hand, some genes of CYP450 family were found upregulated in our BC tissue samples. CYP450-dependent monooxygenases have an essential function in the metabolism of chemical carcinogens. Polymorphisms in CYP450 genes correlate with cancer susceptibility, including BC [20]. Therefore, during carcinogenesis BC cells could enhance antioxidant and detoxifying pathways to control the redox state and to modulate the immune system with the purpose of avoiding apoptosis and enhancing proliferation.

Immunotherapy is currently an area of great interest in BC. In this sense, more insight in the connection between immune system and metabolism could be interesting to understand how BC cells modulate local levels of nutrients to alter immune cell function. Metabolites and transcriptional factors identified as important in our study such as lactate, AAs, fatty acids, GSH and PPARG would have an immunometabolic role in the BC tumor microenvironment. Glutamine uptake is critical for various T-cell metabolic processes (TCA cycle, nucleotide synthesis and detoxification of ROS) [36]. Therefore, in an often-hostile tumor microenvironment, glutamine may be limited and could induce immunosuppression in BC. On the other hand, the role of GSH as anti-inflammatory molecule and its role in processes of innate and adaptive immune system have been also reported [37]. Finally, the function of PPARG has also been linked to the mechanism of action by which BCG inhibits bladder tumor growth [27]. PPARG would repress T-cell effects by means of transrepression of pro-inflammatory processes that result in cytokine production. For all this, PPARG has been proposed as a promising therapeutic target for cancer treatment [38].

In silico analysis of transcriptome data indicated that metabolic genes are mostly downregulated in BC by the action of transcriptional repressors and histone marks. Two examples are GABP and E2F1, already related to bladder carcinogenesis but not with its metabolism. GABP modulates gene expression of housekeeping genes [39] but also would regulate the expression of respiratory chain genes, so it could be a key regulator of energy metabolism in BC. E2F1 has been related with progression of NMIBC to MIBC [40] but according to our results it could regulate several metabolic pathways such as: OXPHOS, TCA cycle, pyruvate, polyamine, and AA metabolism. Particular active and repressive transcription histone marks were also associated with downregulated metabolism-related genes, indicating that epigenetic processes are important in BC regulation. On the other hand, the role of disturbed alternative splicing (AS) promoting pro-tumorigenic isoforms of metabolic enzymes [41] should be considered. We found a significant AS in several metabolic genes in BC tissue samples that may contribute to the observed metabolic changes. The impact of these isoforms on tumor progression and cancer metabolism has not been well studied. Only the role of AS in the gene OGDH has been reported in colorectal cancer, where an up-regulation of OGDH alternative mRNA transcript has been linked with glutamine metabolism [42] and an increase in energy. Splicing variants of GLS gene (KGA and GAC) have been also linked to certain types of tumors [41]. However, the study here presented did not allow for elucidation of the two functional isoforms in BC tissue samples.

Regarding urine samples as a non-invasive alternative to detect BC, our study revealed the presence of several common altered pathways in BC mainly related with the metabolism of taurine and AAs, and also showed their own urinary metabolic pathways, different to those found in the tissue study. The metabolization of some tissue metabolites in other derivatives and the large number of substances present in urine coming from other sources could explain these results. Even so, our data reinforce the idea that some tumor metabolic alterations (disturbed metabolic pathways for alanine, aspartate and glutamate metabolism, taurine and hypotaurine, TCA cycle and aminoacyl t-RNA biosynthesis) are reflected in the urinary metabolome (changes in valine, methylsuccinic acid, lactate, alanine, lysine, N-acetylneuraminic acid, glutamine, succinic acid, citrate, creatinine, trimethylamine-N-oxide, methanol, taurine, sucrose, hippuric acid, phenylalanine, pseudouridine and trigonelline), suggesting that urine can be used to find a non-invasive approach for BC diagnosis and follow-up. Our tissue metabolic profile distinguished BC from non-tumor tissue samples even at early pathologic stage (Ta) with a sensitivity and specificity of 100%. The higher grade of tumors included in the validation set might have enabled a better classification of samples compared to the calibration set. On the other hand, our metabolic urinary signature also showed a high sensitivity (91%) and a good specificity (77%) classifying BC and control urine samples. Considering the limitations of cystoscopy (being expensive, operator-dependent, invasive, and overlooking CIS) and the urinary cytology (low sensitivity in low-grade tumors) the metabolic urinary signature could be used to detect and monitor the dynamic changes in the disease, and for diagnosis and follow-up of BC patients, reducing the number of cystoscopies required in NMIBC patients and identifying early tumor development or recurrences.

## 4. Materials and Methods

### 4.1. Patient Selection and Sample Collection

This study was conducted in accordance with the Declaration of Helsinki, and the protocol was approved by the Ethics Committee for Biomedical Research of the Instituto de Investigación Sanitaria Hospital Universitario y Politécnico La Fe (Valencia, Spain) (approval number 2012/0186). 21 BC patients (14 males and 7 females) were recruited in the urological service of the Hospital Universitario y Politécnico La Fe and they gave their informed consent for inclusion before they participated in the study. All the patients underwent TUR. Tissue and urine samples were collected from each patient. The samples were stored by the Biobanco La Fe (PT13/0010/0026) and processed following standard operating procedures. 

Tumor (n = 22) and adjacent non-tumor tissue samples (n = 22) were collected during TUR, and were immediately placed into cryo-vials, immersed in liquid N2 and stored at −80 °C. Adjacent pieces of the tumor tissue samples underwent routine histopathological examination. BC presence, grade and tumor stage were determined, and tumors were classified as NMIBC (Ta-T1) or MIBC (≥T2). Two tumor episodes (primary tumor and recurrence) were studied from one patient. Therefore, 22 tumor samples were collected from 21 patients.

Urine samples were collected before TUR (pre-TUR: BC, n = 22) and one month after TUR (post-TUR: control, n = 13) and were stored at −80 °C. Nine urine samples post-TUR, from MIBC patients that underwent radical cystectomy and NMIBC with incomplete TUR or taking antibiotics at the time of urine collection, were discarded. BC absence in post-TUR samples was confirmed through cystoscopy. The clinical and demographical data are shown in Table 2, and the scheme of sample collection is shown in Supplementary Figure S1.

### 4.2. Tissue NMR Experiments

Tissue preparation and HRMAS NMR spectra acquisition procedures are described in the Supplementary files. Briefly, 1D ^1^H NOESY spectra were acquired in a 600 MHz spectrometer (Bruker GmbH, Rheinstetten, Germany). 2D homonuclear correlation spectra were acquired in two representative samples to facilitate the signals assignment. After NMR study, the disposable inserts containing the tissue samples were frozen and preserved at −80 °C until the transcriptomic analysis.

### 4.3. Tissue Transcriptomic Experiments

Frozen tissue samples after HRMAS NMR experiments were thawed and underwent microarray experiments. Total RNA was extracted with the miRNeasy Mini Kit, DNA was digested, and RNA integrity checked on an Agilent 2100 Bioanalyzer. After quality filtering, 8 primary tumors (Ta n = 3; T1 n = 2; T2 n = 3) and 10 non-tumor tissue samples were analyzed. cDNAs from total RNA (30 ng) were generated, fragmented, biotinylated, and hybridized to the GeneChip Human Transcriptome Array 2.0 (HTA 2.0) (Affymetrix, Thermofisher, Watham, MA, USA). The arrays were washed and stained on a GeneChip Fluidics Station 450 (Affymetrix); scanning was carried out using the GeneChip Scanner 3000 7G; and image analysis with the AffymetrixGeneChip Command Console software.

### 4.4. Urine NMR Experiments

Urine samples preparation and NMR spectra acquisition procedures are described in the Supplementary Information methods. Briefly, 1D ^1^H NOESY spectra were acquired in a 600 MHz spectrometer (Bruker GmbH, Rheinstetten, Germany) and 2D homonuclear and heteronuclear correlation spectra were acquired in two representative samples to assess signals assignment.

### 4.5. Tissue NMR Data Pre-Processing and Analysis

Spectra processing and handling was performed with MestReNova (version 6.0.2). Spectra were Fourier-transformed, baseline and phase corrected, and chemical shift referenced to the creatine singlet (3.03 ppm) and alanine doublet (1.48 ppm). The main metabolites in the region from 0.8 to 9.5 ppm were assigned, according to the bibliography and NMR [17,43,44,45,46]. Each tissue spectrum was normalized to the sample weight.

Mean metabolite intensities were compared between groups. The intensity of the assigned resonances was transferred to MetaboAnalyst 3.0 [47]. U-Mann–Whitney test determined the significant differences between control and BC tissue samples. ANOVA (Tukey’s post-hoc test) evaluated the differences between NMIBC, MIBC and control tissue, and the changes between Ta, T1, T2 stages and control tissue.

A chemometric analysis was performed on 1D spectra. Regions from 0.5–4.8 and 5.2–9.5 ppm were included. PLS-DA was performed using the software PLS_Toolbox Solo 8.0 (Eigenvector Research, Inc., Manson, WA, USA). Previously, the spectra were peak aligned using Icoshift algorithm [48] to avoid small changes in chemical shift due to differences in pH, and the data were autoscaled. The set of tissue spectra was split up into calibration (n = 34) and validation (n = 10) subsets randomly selected (See Table 1). Two PLS-DA models were calculated including the original set of 11698 spectral features and after a feature selection based on the VIP score using VIP = 1 (4800 features retained) as threshold value. 

The most discriminant metabolites in the PLS-DA model (VIP > 1) were used to perform a pathway enrichment and topology analysis using a global test and a relative betweenness centrality measure in MetaboAnalyst.

### 4.6. Tissue Transcriptomic Data Analysis

The expression of data was normalized and background and batch corrected using the Signal Space Transformation-Robust Multi-Chip Analysis (SST-RMA) implemented in the Transcriptome Analysis Console software version 4.0 (TAC 4.0). Data were deposited in GEO (GSE121711). A PCA and a heatmap analysis were carried out to observe differences between BC and non-tumor tissue samples on the basis of the whole transcriptome. A fold change of at least 2 or −2 and a false discovery rate of 0.05 was considered as selection criteria. GOBP, ChEA and Encode Histone Roadmap were performed in silico using Enrich webtool [18]. Metabolism related genes showing deregulation between tumor and non-tumor samples were selected from GOBP and GeneCards. ChEA and Encode Histone Roadmap analysis were performed to identify the putative transcription factor binding and histone marks in these metabolic genes. Moreover, the identified genes were used to perform non-supervised hierarchical clustering of TCGA data according stage, grade and mRNA subtypes [49] using MeV [50].

Integrated metabolic pathway analysis on results obtained from combined metabolomics (the most important metabolites in the PLS-DA model) and transcriptomics analyses in tissue samples were carried out using the information of: joint pathway analysis of MetaboAnalyst platform, Wikipathways tool, and the Small Molecule Pathway Database (SMPDB) [51].

### 4.7. Urine NMR Data Pre-Processing and Analysis

Spectra were Fourier-transformed, baseline and phase corrected, and the chemical shift was referenced to the DSS (0.0 ppm). The main metabolites in the region from 0.8 to 9.5 ppm were identified, according to already published data and NMR databases [17,43,44,45,46]. The spectra were binned into 0.003 ppm buckets using MestReNova.

A PLS-DA analysis was performed considering the spectral regions from 0.8–4.5 and 6.5–9.0 ppm (see Supplementary files). Previously, urinary spectra were normalized to the sum of all variables and autoscaled. Urine samples were classified as control (post-TUR) or BC (pre-TUR). CV was performed as an approximation to external validation for the estimation of the generalization performance. CV data splitting was performed at the highest level of sampling hierarchy, which in this case was the volunteer. The statistical significance of PLS-DA figures of merit estimated by CV was assessed by permutation test with 100 iterations. The most important metabolites in the statistical model (VIP > 1) were identified and used to perform a pathway enrichment and topology analysis using MetaboAnalyst.

## 5. Conclusions

In summary, our integrated metabolomic and transcriptomic study indicate that metabolic reprogramming in BC is produced mainly through the downregulation of metabolic genes related with TCA cycle, OXPHOS, and AAs metabolic pathways. These tumor tissue metabolic alterations are reflected in the urinary metabolome of the same patient and provide a specific NMR-metabolic profile able to detect BC with elevate sensitivity and specificity from a non-invasive approach.

## Figures and Tables

**Table 1:** Identified metabolites and associated pathways in BC tissue and urine samples.

Metabolic altered Pathways	Sample	Metabolites	*p*	Impact
**Alanine, aspartate, glutamate**	Tissue	Alanine, glutamine, glutamate	6.5E-4	0.44
	Urine	Alanine, glutamine, succinate	6.5E-4	0.26
**Taurine and hypotaurine**	Tissue	Taurine, alanine	9.0E-3	0.36
	Urine	Taurine, alanine	9.0E-3	0.36
**Aminoacyl-tRNA biosynthesis**	Tissue	Proline, glycine, alanine, glutamine, threonine, glutamate	1.0E-5	0.06
	Urine	Phenylalanine, glutamine, valine, alanine, lysine	1.6E-4	0.56
**Methane**	Tissue	Glycine, methanol	2.6E-2	0.02
	Urine	Trimethylamine N-oxide, methanol	2.5E-2	0.02
**Glycine, serine, threonine**	Tissue	Glycine, creatine, choline, threonine	3.5E-4	0.29
**Glutathione**	Tissue	Glutathione, glycine, glutamate	2.0E-3	0.25
**Glycerolipid**	Tissue	Fatty acid, glycerol	2.3E-2	0.19
**Arginine and proline**	Tissue	Glutamine, glutamate, proline, creatine	2.0E-3	0.17
**Glutamine and glutamate**	Tissue	Glutamine, glutamate	3.0E-3	0.14
**Glycerophospholipid**	Tissue	Choline, glycerophosphocholine, phosphocholine	3.0E-3	0.08
**Citrate cycle**	Urine	Citrate, succinate	9.0E-3	0.08
**Phenylalanine**	Urine	Succinate, phenylalanine, hippuric acid	4.0E-3	0.07
**Nitrogen metabolism**	Urine	Phenylalanine, taurine, glutamine	2.7E-3	0.05
**Propanoate**	Urine	Succinate, lactic acid, valine	2.0E-3	0.05

**Table 2:** Clinical and demographic data of patients included in this study.

	Tissue Samples		Urine Samples
PLS-DA Models (BC *vs* Control)	Calibration (CV)	Validation	Calibration (CV)
**Patients** (male/female)	17(10/7)	5 (4/1)	21 (14/7)
**Age** (mean and standard deviation)	71 (9)	63 (11)	69 (10)
**Samples** (male/female)	34 (20/14	10 (8/2)	35 (23/11)
**Tumor samples** (BC)	17	5	22
**Non-tumor samples** (Control)	17	5	13
**Primary/Recurrent BC**	15/2	4/1	19/3
**Tumor stage** (Ta, T1, T2)	7/6/4	3/0/2	10/6/6
**Tumor grade** (1/2/3)	4/3/10	0/2/3	18/4
**Recurrence risk group 5 yr (EORTC)**^**a**^: L/ L-I/ H-I/ H	2/4/6/1	0/0/3/0	2/4/9/1
**Progression risk group 5 yr (EORTC) **^**a**^: L/L-I/H-I/H	4/2/5/2	0/1/2/0	4/3/7/2

NOTE: ^a^: Probability of recurrence and progression in NMIBC (n = 16) according to the EORTC risk tables total score; L: Low risk, L-I: Low-Intermediate risk, H-I: High-Intermediate risk, H: High risk.

## Supplementary Materials

The following are available online at https://www.mdpi.com/2072-6694/11/5/686/s1, Supplementary Materials is provided in Supplementary information file.
