# Supplementary material for: Integrative Metabolomic and Transcriptomic Analysis for the Study of Bladder Cancer
Source: Cancers (Basel). 2019 May 16;11(5):686. doi: 10.3390/cancers11050686 (PMC6562847; doi:10.3390/cancers11050686)
Supplement: Supplementary file 1 [file cancers-11-00686-s001.pdf]

cancers

Supplemental Materials

Integrative Metabolomic and Transcriptomic Analysis for the Study of Bladder Cancer

Alba Loras, Cristian Suárez-Cabrera, M. Carmen Martínez-Bisbal, Guillermo Quintás, Jesús M. Paramio, Ramón Martínez-Máñez, Salvador Gil and José Luis Ruiz-Cerdá

Supplemental methods

Tissue preparation for ^{1}H HRMAS NMR spectroscopy: Tissue were prepared for measurements according to already published data [1]. Briefly, each sample was weighted. The amount of tumor tissue analyzed for each patient ranged from 8.7 to 37.8 mg. Each sample was introduced into a disposable KelF insert with an external diameter of 3 mm and an internal diameter of 2.6 mm (Bruker, Billerica, MA, USA). The total internal volume of the insert was about 30 µL. 10 µL of D2O was added to each insert and it was sealed with a small screw cap.

Tissue spectra acquisition by ^{1}H HRMAS NMR spectroscopy: The insert containing the bladder tissue was put in a standard 4mm ZrO2 rotor, and this was transferred to the NMR probe, which was precooled at 0 °C. Experiments were acquired at a real temperature of 277 °K. Tissue spectra were acquired using a Bruker Avance DRX 600 spectrometer (Bruker GmbH, Rheinstetten, Germany) operating at a ^{1}H frequency of 600.13 MHz. The instrument was equipped with a 4 mm triple resonance $^{1}H/^{13}C/^{15}N$ HRMAS probe with magnetic field gradients aligned with the magic angle axis. A Bruker cooling unit was used to control the temperature by cooling down the bearing air flowing into the probe. For all NMR experiments, samples were spun at 5 kHz in order to keep the rotation sidebands out of the acquisition window. For each tissue sample, one-dimensional (1D) ^{1}H NOESY spectrum with water presaturation was acquired in 5 min using a 1.14 s acquisition time, 128 transients, a 12 ppm (7211 Hz) spectral width, a mixing time of 100 ms and a relaxation delay of 1 s. In addition, two-dimensional (2D) ^{1}H-^{1}H TOCSY using a DIPSI2 sequence for mixing were acquired with a 142 ms acquisition time, 50 ms spin lock duration, 7211 Hz spectral width, and a 1.5 s relaxation delay. Sixteen transients were averaged for each of the 256 increments during t1, corresponding to a total acquisition time of 2 h 33 min.

Urine sample preparation for ^{1}H NMR spectroscopy: Urine samples were thawed at room temperature and were prepared following the established procedures for urine samples [2]. Phosphate buffer (pH 7.4) was prepared by weighing Na2HPO4 (28.85 g), NaH2PO4 (5.25 g), NaN3 (0.195 g) and DSS (0.218 g) into a 1 L volumetric flask. 200 mL of D2O were added and the flask was filled to 1 l with water. 500 µL of urine were added 200 µL of phosphate buffer. This mix was centrifuged at 10.000 rpm for 5 min at 5 °C. After this, 550 µL of the supernatant were transferred to a 5 mm NMR tube for analysis.

Urine spectra acquisition by ^{1}H NMR spectrometry: The experiments were recorded at 298 °K. Spectra were acquired using a Bruker Avance DRX 600 spectrometer (Bruker GmbH, Rheinstetten, Germany) operating at a ^{1}H frequency of 600.13 MHz. The instrument was equipped with a 5mm triple resonance $^{1}H/^{13}C/^{31}P$ probe. For each urine sample, 1D ^{1}H NOESY spectra using water presaturation were acquired in 3 min using a 3.91 s acquisition time, 32 transients, a 14 ppm (8370 Hz) spectral width, a mixing time of 100 ms and a relaxation delay of 1 s. Moreover, 2D ^{1}H-^{13}C HSQC spectra were acquired, to assess the assignments of the overlapped signals in 1D ^{1}H spectra.

Data pre-processing and statistical analysis: After spectra acquisition, the free induction decay (FID)'s was Fourier transformed, phase and baseline corrected and chemical shift referenced with MestReNova version 6.0.2 (Mestrelab Research SL, Santiago de Compostela, Spain).

In tissue samples, chemical shift was referenced to creatine (CH3) singlet 3.03 ppm and to alanine (CH3) doublet 1.48 ppm and peak alignment was performed after chemical shift referencing

using Icoshift algorithm [3]. Entire spectrum was divided into 178 segments and each segment was aligned separately. For chemometrics analysis the chemical shift spectral region of 0.5–9.5 ppm was considered. Spectral region of water (4.79–5.2 ppm) was excluded. Tissue spectra were normalized to their weight (mg).

PCA and PLS-DA were performed using the software PLS_Toolbox Solo 8.0 (Eigenvector Research, Inc., Manson, WA, USA). Before PCA and PLS-DA analysis, data were autoscaled. This pre-processing method uses mean-centering followed by division of each column (variable) by the standard deviation of that column. The number of LVs was selected according to the minimum root mean square error for cross-validation (RMSECV). The selection of the VIP threshold values was based on the analysis of the evolution of CV-figures of merit (dQ_2 and mean classification error of CV) as a function of the threshold value. Features election during model optimization aimed at improving the predictive model performance and facilitating its interpretation.

In urine samples, the chemical shift was referenced to 4,4-dimethyl-4-silapentane-1-sulfonic acid (DSS at known concentration) at 0.0 ppm and 1D ^{1}H NOESY spectra were binned into 0.003 ppm buckets using MestReNova. The chemical shift spectral region (0.8–4.5 6.5–9.0 ppm) were considered for the statistical analysis. Water (4.5–5.1 ppm), urea (5.5–6.1 ppm) and regions lower 0.8 ppm and greater 9.00 ppm were excluded from the analysis to avoid interferences arising from differences in water suppression and variability from urea and DSS signals. The number of LVs was selected according to the RMSECV. PCA and PLS-DA were performed using PLS_Toolbox Solo 8.0 before PCA and PLS-DA analysis data were normalized to the sum of all data and were autoscaled.

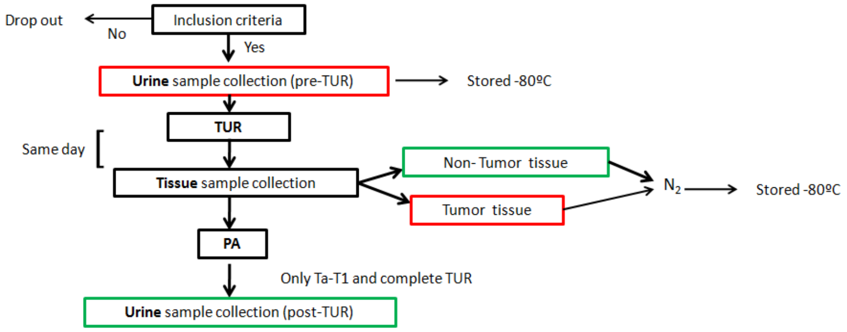

Figure S1. Sample collection scheme. NOTE: PA: Pathological Anatomy; TUR: transurethral resection.

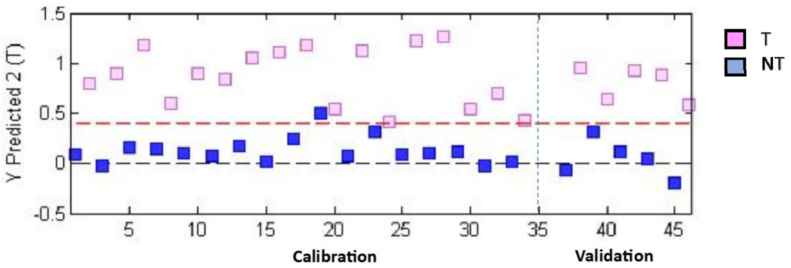

Figure S2. Predicted y values for calibration and validation subsets considering the PLS-DA model performed in bladder tissues after an initial feature selection (VIP>1).

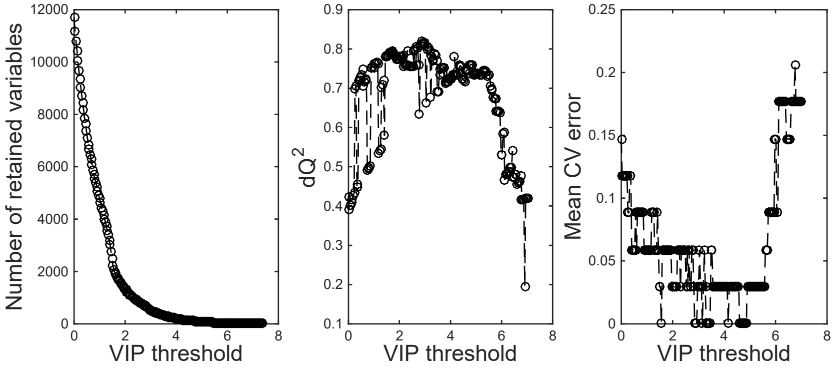

Figure S3. Evolution of three indicators (number of features, discriminant Q^2 and mean CV-classification error) of the discriminant performance of the PLS-DA model as a function of the VIP cutoff value used for the elimination of features in an initial PLS-DA model.

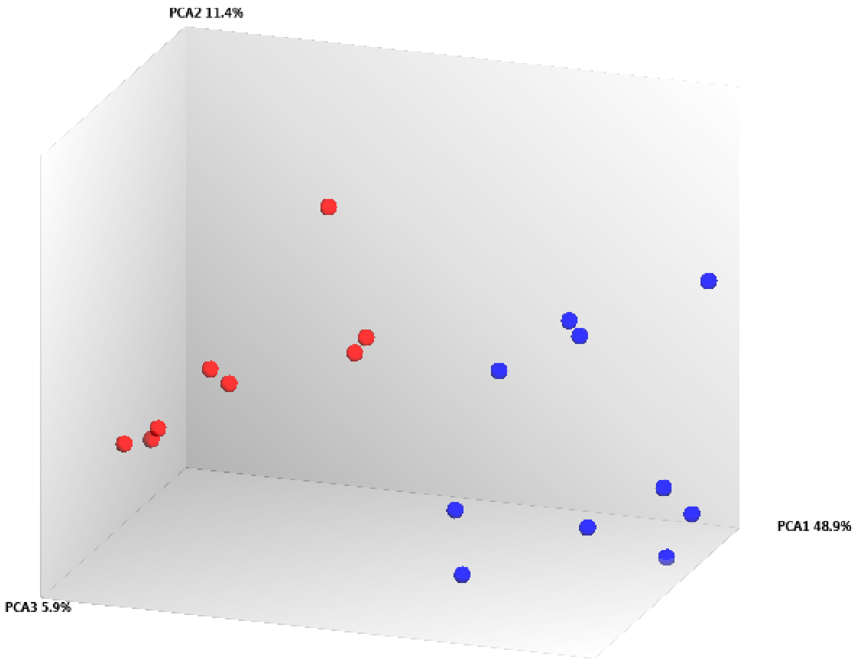

Figure S4. Principal Component Analysis (PCA) using the overall transcriptome. NOTE: Tumors are shown in red and non-tumors in blue.

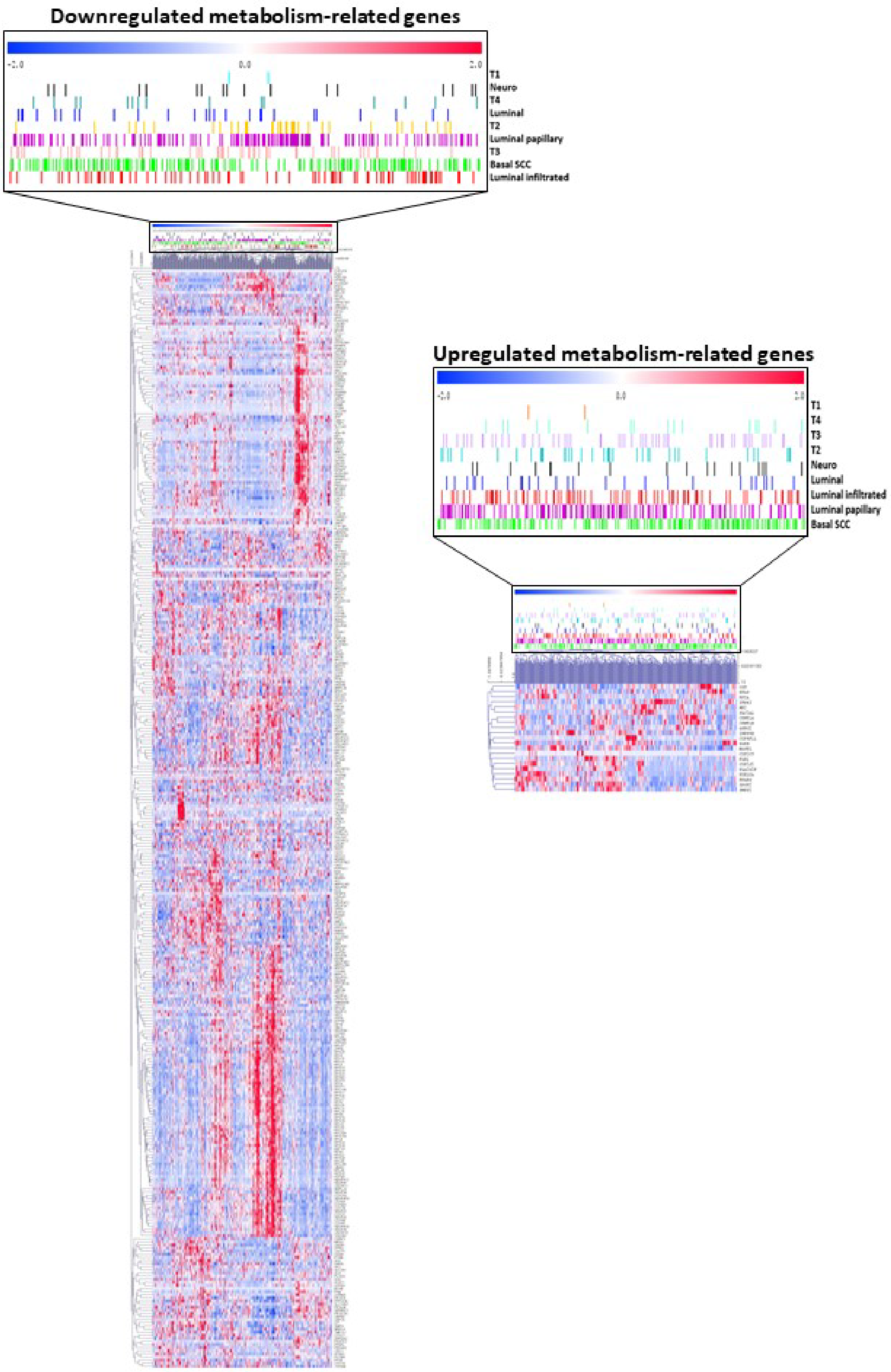

Figure S5. Comparison between upregulated and downregulated metabolism-related genes with gene expression of tumor samples present in the TCGA cohort.

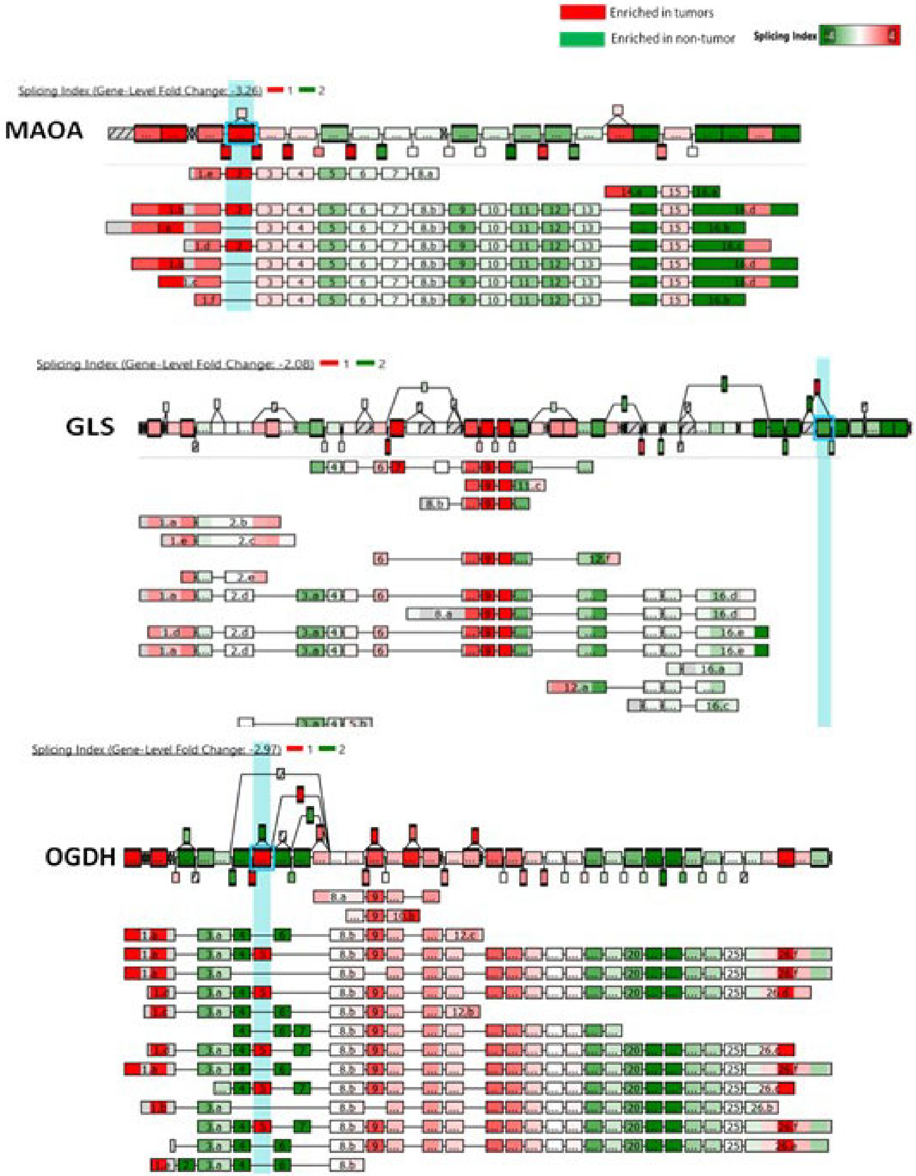

Figure S6. Splicing variant analyses in some metabolic genes. NOTE: *MAOA*: Monoamine Oxidase A; *GLS*: Glutaminase; *OGDH*: Oxoglutarate Dehydrogenase.

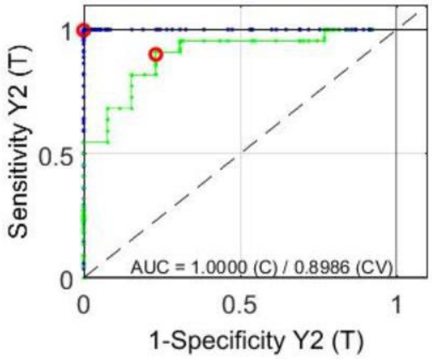

Figure 7. Receiver operating characteristic (ROC) calculated for cross validation through a PLS-**DA model performed in urine samples.**

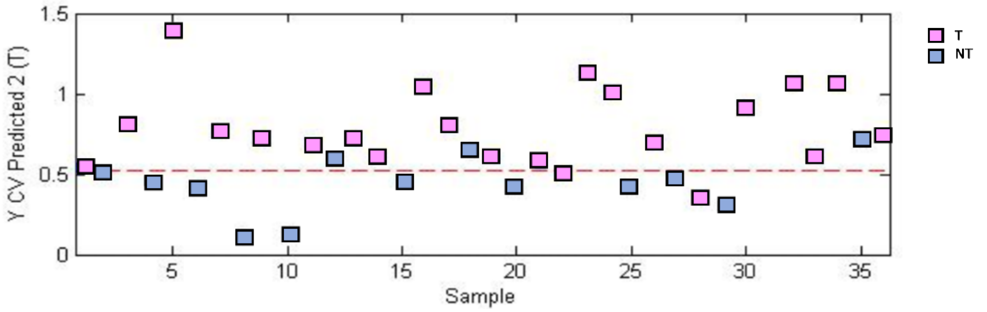

Figure S8. Predicted *y* values for CV considering the PLS-DA model performed in bladder urines.

Table S1. Assignment of the main metabolites identified in NMR tissue spectra.

N. Assigment	Metabolite	Group	Chemical shift (ppm)
1.1	Lipid fragment –(n)CH_2-CH_2-CH_2-CH_3 (a)	CH_3	0.90
2	Leucine	δCH_3	0.93
3	Valine	γCH_3	0.98
3	Valine	γCH_3	1.04
1.1	Lipid fragment –(n)CH_2-CH_2-CH_2-CH_3 (a)	(n) CH_2	1.29
1.1	Lipid fragment –(n)CH_2-CH_2-CH_2-CH_3 (a)	(2) CH_2	1.29
1.2	Lipid fragment –CH=CH-CH_2-CH=CH-CH_2-CH_2-(n)CH_2 (b)	(n) CH_2	1.29
1.3	Lipid fragment –(n)CH_2-CH_2-CH_2-COOH (c)	(n) CH_2	1.29
1.1	Lipid fragment –(n)CH_2-CH_2-CH_2-CH_3 (a)	(1) CH_2	1.33
1.2	Lipid fragment –CH=CH-CH_2-CH=CH-CH_2-CH_2-(n)CH_2 (b)	(1) CH_2	1.33
4	Lactate	CH_3	1.33
5	Threonine	γCH_3	1.33
6	Alanine	βCH_3	1.46
1.3	Lipid fragment –(n)CH_2-CH_2-CH_2-COOH (c)	(2) CH_2	1.59
8	Arginine	γCH_2	1.68
2	Leucine	βCH_2	1.70
2	Leucine	γCH	1.70
7	Lysine	βCH_2	1.71
8	Arginine	βCH_2	1.90
1.2	Lipid fragment –CH=CH-CH_2-CH=CH-CH_2-CH_2-(n)CH_2 (b)	(2) CH_2	2.03
9	Glutamate	βCH_2	2.05
11	Proline	βCH_2	2.06
12	Glutamine	βCH_2	2.13
10	Glutathione	CH_2	2.15
1.3	Lipid fragment –(n)CH_2-CH_2-CH_2-COOH (c)	(1) CH_2	2.26
9	Glutamate	γCH_2	2.33
11	Proline	βCH_2	2.34
12	Glutamine	γCH_2	2.44
10	Glutathione	CH_2	2.54
13	Creatine	CH_3	3.03
14	Choline	$-N^{+}-(CH3)_3$	3.18
15	Phosphocholine	$-N^{+}-(CH3)_3$	3.20
16	Glycerophosphocholine	$-CH_2-NH_3^{+}$	3.21
17	Taurine	$-CH_2-NH_3^{+}$	3.25
18	Methanol	CH_3	3.34

17	Taurine	-CH_2-SO_3^-	3.42
19	Myo-inositol	$C_{1',3'}$ - H	3.52
20	Glycine	αCH	3.55
19	Myo-inositol	$C_{4',6'}$ - H	3.62
7	Lysine	αCH	3.74
21	Glycerol	-CH-(OH)-	3.78
13	Creatine	CH_2	3.92
4	Lactate	CH	4.10
1.2	Lipid fragment –CH=CH-CH_2-CH=CH-CH_2-CH_2-(n)CH_2 (b)	=CH-	5.30
22	UDP-sugars	__	5.99
23	Tyrosine	$C_{3',5'}$ - H	6.89
23	Tyrosine	$C_{2',6'}$ - H	7.17
24	Phenylalanine	$C_{2',6'}$ - H	7.32
24	Phenylalanine	$C_{3',5'}$ - H	7.42
25	Cytidine diphosphate	CH	7.97
10	Glutathione	NH	8.15

Table S2. Significant metabolites in bladder tissues using U-Mann-Whitney test between tumor and non-tumor tissues (Table S2-A); ANOVA test among NMIBC, MIBC and CTRL tissues (Table S2-B); and ANOVA test among Ta, T1, T2 and CTRL tissues (Table S2-C).

Table S2-A

Mol. Fmla.	Metabolites	HMDB	*p*-value	T/NT
--	UDP-sugars	--	<0.05	>1
$C_9H_{15}N_3O_{11}P_2$	Cytidine diphosphate	HMDB01546	<0.05	>1
$C_{10}H_{17}N_3O_6S$	Glutathione	HMDB00125	<0.05	>1
$C_5H_{14}NO$	Choline	HMDB00097	<0.05	>1
$C_6H_{12}O_6$	Myo-inositol	HMDB00211	<0.05	>1
–(n)CH_2-CH_2-CH_2-COOH	Lipid (c)	--	<0.05	<1

Table S2-B

Mol. Fmla.	Metabolites	HMDB	*p*-value	Tukey's Post-hoc tests
--	UDP-sugars	--	<0.05	MIBC:CTRL NMIBC:CTRL
$C_9H_{15}N_3O_{11}P_2$	Cytidine diphosphate	HMDB01546	<0.05	MIBC:CTRL NMIBC:CTRL
$C_{10}H_{17}N_3O_6S$	Glutathione	HMDB00125	<0.05	MIBC:CTRL NMIBC:CTRL
$C_5H_{14}NO$	Choline	HMDB00097	<0.05	MIBC:CTRL NMIBC:CTRL
$C_6H_{12}O_6$	Myo-inositol	HMDB00211	<0.05	NMIBC:CTRL

Table S2-C

Mol. Fmla.	Metabolites	HMDB	*p*-value	Tukey's Post-hoc tests
$C_5H_{14}NO$	Choline	HMDB00097	<0.05	T2:T1 T2:Ta T1:Ta T2:CTRL T1:CTRL Ta:CTRL
$C_9H_{11}NO_3$	L-Tyrosine	HMDB0000158	<0.05	T2:T1 T2:Ta T1:Ta T2:CTRL T1:CTRL Ta:CTRL
$C_6H_{12}O_6$	Myo-inositol	HMDB00211	<0.05	T2:T1 T2:Ta T1:Ta T2:CTRL T1:CTRL Ta:CTRL
$C_{10}H_{17}N_3O_6S$	Glutathione	HMDB00125	<0.05	T2:T1 T2:Ta T1:Ta T2:CTRL T1:CTRL Ta:CTRL
–(n)CH_2-CH_2-CH_2-CH_3	Lipid (a)	--	<0.05	T2:T1 T2:Ta T1:Ta T2:CTRL T1:CTRL Ta:CTRL
---	UDP-sugars	--	<0.05	T2:T1 T2:Ta T1:Ta T2:CTRL

$C_9H_{15}N_3O_{11}P_2$	Cytidine diphosphate	HMDB01546	<0.05	T1:CTRL Ta:CTRL T2:T1 T2:Ta T1:Ta T2:CTRL T1:CTRL Ta:CTRL
–CH=CH-CH_2-CH=CH -CH_2-CH_2-(n)CH_2-	Lipid (b)	--	<0.05	T2:T1 T2:Ta T1:Ta T2:CTRL T1:CTRL Ta:CTRL

NOTE: HMDB: Human Metabolome Database; Mol.Fmla: Molecular formula; T/NT: ratio mean value in tumor over non-tumor samples.

Table 3. Indices of test validity estimated for the evaluation of predictive performance of PLS-DA models between tumor and non-tumor tissue samples with LVs = 2. PLS-DA model using all features = 11698.

PLS-DA model	CV	Validation
**Sensitivity**	82.4 (62.3–97.9)%	100 (46.3–98.1)%
**Specificity**	88.2 (55.8–95.3)%	100 (46.3–98.1)%
**PPV** [a]	87.5 (60.4–97.8)%	100 (46.3–98.1)%
**NPV** [b]	83.3 (57.7–95.6)%	100 (46.3–98.1)%
**ACC** [c]	85.3%	100%
**MCC** [d]	0.707	1
**PT** [e] **(p-value)**	--	0.015
**AUROC**	--	1

NOTE: CV: Cross validation; [a] Positive predictive value; [b] Negative predictive value; [c] Diagnostic accuracy; [d] Matthew's correlation coefficient; [e] Permutation test).

Table S4. Metabolic genes significantly altered in bladder tumors.

Gene	Description	Regulation	FC	*p*-value	FDR p-val
AASS	aminoadipate-semialdehydesynthase	DOWN	−2.55	2.00E-03	8.90E-03
ABL1	ABL proto-oncogene 1, non-receptor tyrosinekinase	DOWN	−5.80	6.63E-06	2.00E-04
ACAA2	acetyl-CoAacyltransferase 2	DOWN	−5.13	2.10E-06	6.80E-05
ACACB	acetyl-CoAcarboxylase beta	DOWN	−8.43	5.27E-07	2.36E-05
ACADM	acyl-CoAdehydrogenase, C-4 to C-12 straightchain	DOWN	−2.31	1.95E-07	1.12E-05
ACAT1	acetyl-CoAacetyltransferase 1	DOWN	−2.60	4.68E-06	1.00E-04
ACO1	aconitase 1, soluble	DOWN	−4.27	1.38E-08	1.54E-06
ACSL3	acyl-CoAsynthetase long-chainfamilymember 3	DOWN	−2.09	8.00E-04	4.70E-03
ADARB1	adenosine deaminase, RNA-specific, B1	DOWN	−14.27	1.67E-07	9.90E-06
ADH1B	alcohol dehydrogenase 1B (class I), beta polypeptide	DOWN	−97.23	4.94E-06	1.00E-04
ADH1C	alcohol dehydrogenase 1C (class I), gamma polypeptide	DOWN	−3.84	7.78E-06	2.00E-04
ADH5	alcohol dehydrogenase 5 (class III), chi polypeptide	DOWN	−3.58	1.65E-10	9.70E-08

AK2	adenylate kinase 2	DOWN	−2.40	1.00E-04	1.30E-03
AK4	adenylate kinase 4	DOWN	−12.83	1.25E-06	4.61E-05
ALDH1B1	aldehyde dehydrogenase 1 family, member B1	DOWN	−7.33	3.33E-08	2.85E-06
ALDH2	aldehyde dehydrogenase 2 family (mitochondrial)	DOWN	−5.62	1.19E-07	7.65E-06
ALDH6A1	aldehyde dehydrogenase 6 family, member A1	DOWN	−2.39	2.47E-07	1.32E-05
ALDH9A1	aldehyde dehydrogenase 9 family, member A1	DOWN	−2.04	1.31E-09	3.21E-07
AMD1	adenosylmethionine decarboxylase 1	DOWN	−2.13	4.00E-04	3.10E-03
AOC3	amineoxidase, copper containing 3	DOWN	−6.48	7.01E-07	2.98E-05
APLP2	amyloid beta (A4) precursor-like protein 2	DOWN	−5.67	9.07E-05	1.10E-03
APP	amyloid beta (A4) precursor protein	DOWN	−4.43	7.61E-07	3.15E-05
ASS1	argininosuccinate synthase 1	DOWN	−2.07	2.23E-05	4.00E-04
ATIC	5-aminoimidazole-4-carboxamide ribonucleotide formyl transferase/IMP cyclohydrolase	DOWN	−2.48	4.57E-06	1.00E-04
ATP5A1	ATP synthase, H+ transporting, mitochondrial F1 complex, alphasubunit 1, cardiac muscle	DOWN	−2.18	1.84E-05	3.00E-04
ATP5B	ATP synthase, H+ transporting, mitochondrial F1 complex, beta polypeptide	DOWN	−9.72	4.72E-09	7.48E-07
ATP5C1	ATP synthase, H+ transporting, mitochondrial F1 complex, gamma polypeptide 1	DOWN	−3.83	2.29E-07	1.26E-05
ATP5D	ATP synthase, H+ transporting, mitochondrial F1 complex, delta subunit	DOWN	−2.95	2.39E-09	4.62E-07
ATP5E	ATP synthase, H+ transporting, mitochondrial F1 complex, epsilonsubunit	DOWN	−4.53	1.55E-06	5.43E-05
ATP5F1	ATP synthase, H+ transporting, mitochondrial Fo complex subunit B1	DOWN	−2.05	3.19E-08	2.77E-06
ATP5G2	ATP synthase, H+ transporting, mitochondrial Fo complex subunit C2 (subunit 9)	DOWN	−41.60	4.29E-10	1.63E-07
ATP5G3	ATP synthase, H+ transporting, mitochondrial Fo complex subunit C3 (subunit 9)	DOWN	−19.75	1.29E-08	1.48E-06
ATP5H	ATP synthase, H+ transporting, mitochondrial Fo complex subunit D	DOWN	−4.29	7.01E-06	2.00E-04
ATP5I	ATP synthase, H+ transporting, mitochondrial Fo complex subunit E	DOWN	−10.48	3.33E-08	2.85E-06
ATP5J	ATP synthase, H+ transporting, mitochondrial Fo complex subunit F6	DOWN	−2.06	1.20E-08	1.40E-06
ATP5L	ATP synthase, H+ transporting,	DOWN	−2.31	1.00E-04	1.30E-

	mitochondrial Fo complex subunit G				03
ATP5O	ATP synthase, H+ transporting, mitochondrial F1 complex, O subunit	DOWN	−17.06	2.97E-08	2.62E-06
ATP6AP1	ATPase, H+ transporting, lysosomalaccessoryprotein 1	DOWN	−6.97	6.39E-05	8.00E-04
ATP6V0A1	ATPase, H+ transporting, lysosomal V0 subunit a1	DOWN	−2.78	2.80E-05	5.00E-04
ATP6V0E1	ATPase, H+ transporting, lysosomal 9kDa, V0 subunit e1	DOWN	−6.95	4.73E-08	3.71E-06
ATP6V1E1	ATPase, H+ transporting, lysosomal 31kDa, V1 subunit E1	DOWN	−2.32	1.82E-07	1.06E-05
ATP6V1F	ATPase, H+ transporting, lysosomal 14kDa, V1 subunit F	DOWN	−3.98	3.16E-06	9.25E-05
ATP8B2	ATPase, aminophospholipidtransporter, class I, type 8B, member 2	DOWN	−2.10	5.04E-05	7.00E-04
AUH	AU RNA bindingprotein/enoyl-CoAhydratase	DOWN	−2.39	7.82E-05	1.00E-03
B2M	beta-2-microglobulin	DOWN	−2.36	3.30E-02	6.82E-02
BACE1	beta-site APP-cleavingenzyme 1	DOWN	−2.58	5.00E-04	3.30E-03
BCHE	butyrylcholinesterase	DOWN	−3.66	7.93E-06	2.00E-04
C3	complement component 3	DOWN	−2.35	6.20E-03	1.94E-02
C4A	complement component 4A (Rodgersbloodgroup)	DOWN	−4.18	1.30E-03	6.50E-03
CALM2	calmodulin 2 (phosphorylasekinase, delta)	DOWN	−9.27	1.25E-10	8.38E-08
CALM3,2,1	calmodulin 3); calmodulin 1); calmodulin 2	DOWN	−7.15	2.87E-09	5.23E-07
CALU	calumenin	DOWN	−5.32	2.83E-07	1.47E-05
CAT	catalase	DOWN	-3.44	1.88E-05	4.00E-04
CDC42BPA	CDC42 binding protein kinase alpha (DMPK-like)	DOWN	-5.89	3.74E-09	6.25E-07
CHPT1	choline phosphotransferase 1	DOWN	-22.00	7.78E-11	6.49E-08
CHRDL1	chordin-like 1	DOWN	-3.73	1.00E-04	1.40E-03
CHRM2	cholinergic receptor, muscarinic 2	DOWN	-4.70	3.00E-06	8.90E-05
CHRM3	cholinergic receptor, muscarinic 3	DOWN	-12.79	1.47E-06	5.21E-05
CIT; MIR1178	citron rho-interacting serine/threonine kinase; microRNA 1178	UP	2.45	2.70E-05	5.00E-04
CKB	creatine kinase, brain	DOWN	-2.95	4.32E-06	1.00E-04
CKMT1A; CKMT1B	creatine kinase, mitochondrial 1A; creatine kinase, mitochondrial 1B	UP	2.77	8.00E-04	4.60E-03
COA5	cytochrome c oxidase assembly factor 5	DOWN	-2.09	6.00E-04	4.00E-

					03
COX10	COX10 heme A:farnesyl transferase cytochrome c oxidase assembly factor	DOWN	-2.57	5.22E-07	2.35E-05
COX11	COX11 cytochrome c oxidase copper chaperone	DOWN	-5.49	6.72E-09	9.62E-07
COX15	cytochrome c oxidase assembly homolog 15 (yeast)	DOWN	-2.67	3.25E-08	2.81E-06
COX20	COX20 cytochrome c oxidase assembly factor	DOWN	-4.40	1.45E-08	1.58E-06
COX4I1	cytochrome c oxidase subunit IV isoform 1	DOWN	-4.84	3.14E-11	5.04E-08
COX5A	cytochrome c oxidase subunit Va	DOWN	-3.13	1.50E-07	9.14E-06
COX5B	cytochrome c oxidase subunitVb	DOWN	-5.14	1.28E-07	8.10E-06
COX6A1	cytochrome c oxidase subunit VI apolypeptide 1	DOWN	-4.01	2.00E-04	2.00E-03
COX6C	cytochrome c oxidase subunit VIc	DOWN	-2.31	1.51E-08	1.63E-06
COX7A1	cytochrome c oxidase subunit VII apolypeptide 1 (muscle)	DOWN	-8.45	3.59E-10	1.52E-07
COX7A2	cytochrome c oxidase subunit VII apolypeptide 2 (liver)	DOWN	-5.00	2.93E-08	2.61E-06
COX7B	cytochrome c oxidase subunitVIIb	DOWN	-4.85	1.94E-07	1.12E-05
COX7C; MIR3607	cytochrome c oxidase subunitVIIc; microRNA 3607	DOWN	-3.74	9.68E-10	2.71E-07
COX8A	cytochrome c oxidase subunit VIIIA (ubiquitous)	DOWN	-82.96	5.73E-11	5.92E-08
CRYM	crystallinmu	DOWN	-2.56	8.16E-07	3.33E-05
CSRP2	cysteine and glycine-richprotein 2	DOWN	-8.27	4.03E-08	3.32E-06
CST3	cystatin C	DOWN	-2.44	2.07E-07	1.16E-05
CYCS	cytochrome c, somatic	DOWN	-2.44	1.20E-03	6.10E-03
CYP1B1	cytochrome P450, family 1, subfamily B, polypeptide 1	DOWN	-2.10	4.10E-03	1.45E-02
CYP2C9	cytochrome P450, family 2, subfamily C, polypeptide 9	UP	2.18	2.00E-04	1.60E-03
CYP2J2	cytochrome P450, family 2, subfamily J, polypeptide 2	UP	7.98	1.59E-02	3.85E-02
CYP4F11	cytochrome P450, family 4, subfamily F, polypeptide 11	UP	2.03	2.52E-02	5.44E-02
CYP4V2	cytochrome P450, family 4, subfamily V, polypeptide 2	DOWN	-2.68	4.61E-08	3.65E-06
CYR61	cysteine-rich, angiogenic inducer, 61	DOWN	-4.12	1.27E-02	3.25E-02
DARS	aspartyl-tRNA synthetase	DOWN	-2.27	1.18E-05	3.00E-04
DGKB	diacylglycerol kinase, beta 90kDa	DOWN	-2.95	3.00E-04	2.30E-

					03
DGKG	diacylglycerol kinase gamma	DOWN	-3.82	1.65E-06	5.69E-05
DGKH	diacylglycerol kinase, eta	UP	-2.12	3.14E-02	6.45E-02
DLAT	dihydrolipoamide S-acetyltransferase	DOWN	-2.03	1.01E-06	3.92E-05
DLD	dihydrolipoamide dehydrogenase	DOWN	-3.22	1.31E-05	3.00E-04
DNAJC3	DnaJ (Hsp40) homolog, subfamily C, member 3	DOWN	-11.87	1.51E-07	9.20E-06
DPYD	dihydropyrimidine dehydrogenase	DOWN	-2.67	2.00E-04	1.90E-03
ECH1	enoyl-CoA hydratase 1, peroxisomal	DOWN	-5.42	6.34E-07	2.74E-05
ECI2	enoyl-CoA delta isomerase 2	DOWN	-3.14	1.12E-08	1.34E-06
EEF1A1	eukaryotic translation elongation factor 1 alpha 1	DOWN	-4.04	4.33E-07	2.03E-05
EEF1G	eukaryotic translation elongation factor 1 gamma; microRNA 3654	DOWN	-4.68	1.95E-05	4.00E-04
EIF4EBP2	eukaryotic translation initiation factor 4E bindingprotein 2	DOWN	-2.19	8.25E-05	1.00E-03
ENTPD1	Ectonucleoside triphosphate diphosphohydrolase 1	DOWN	-9.40	6.96E-09	9.80E-07
ESD	esterase D	DOWN	-8.14	4.76E-10	1.73E-07
FARSB	phenylalanyl-tRNA synthetase beta subunit	DOWN	-2.91	5.27E-07	2.36E-05
FAXDC2	fatty acid hydroxylase domain containing 2	DOWN	-5.15	3.24E-12	1.37E-08
FBN1	fibrillin 1	DOWN	-3.44	1.00E-04	1.40E-03
FH	fumarate hydratase	DOWN	-2.79	4.34E-09	7.01E-07
FN1	fibronectin 1	DOWN	-55.97	7.47E-08	5.30E-06
FNBP1	formin bindingprotein 1	DOWN	-11.83	1.62E-09	3.68E-07
FSTL1	Follistatin like 1; microRNA 198	DOWN	-3.21	3.89E-06	1.00E-04
GAPDH	glyceraldehyde-3-phosphate dehydrogenase	DOWN	-3.10	1.32E-05	3.00E-04
GAS6	growth arrest-specific 6	DOWN	-2.80	7.92E-08	5.56E-06
GATA6	GATA bindingprotein 6	DOWN	-3.50	1.20E-05	3.00E-04
GATM	glycine amidino transferase (L-arginine:glycine amidino transferase)	DOWN	-6.37	1.00E-04	1.30E-03
GLRX3	glutaredoxin 3	DOWN	-2.09	9.45E-05	1.10E-03
GLS	glutaminase	DOWN	-2.08	1.03E-06	3.94E-

					05
GMPS	guanine monophosphate synthase	DOWN	-2.67	2.48E-05	4.00E-04
GNPDA2	glucosamine-6-phosphate deaminase 2	DOWN	-2.41	9.93E-07	3.86E-05
GOT2	glutamic-oxaloacetic transaminase 2, mitochondrial	DOWN	-3.15	5.58E-06	1.00E-04
GPX3	glutathione peroxidase 3	DOWN	-2.77	7.20E-07	3.03E-05
GPX4	glutathione peroxidase 4	DOWN	-2.83	2.77E-08	2.50E-06
GSN	gelsolin	DOWN	-3.52	2.88E-07	1.49E-05
GSTA4	glutathione S-transferase alpha 4	DOWN	-2.60	8.28E-07	3.37E-05
GSTO1	glutathione S-transferase omega 1	DOWN	-2.20	2.32E-08	2.19E-06
GSTO2	glutathione S-transferase omega 2	UP	2.38	5.20E-03	1.71E-02
GSTT1	glutathione S-transferase theta 1	DOWN	-3.00	2.20E-03	9.50E-03
H3F3A	H3 histone, family 3A	DOWN	-3.60	1.00E-04	1.20E-03
HADHA	hydroxyacyl-CoAdehydrogenase/3-ketoacyl-CoA thiolase/enoyl-CoA hydratase , alpha subunit	DOWN	-2.46	1.78E-05	3.00E-04
HADHB	hydroxyacyl-CoA dehydrogenase/3-ketoacyl-CoA thiolase/enoyl-CoA hydratase , beta subunit	DOWN	-2.49	3.00E-04	2.60E-03
HIBADH	3-hydroxyisobutyrate dehydrogenase	DOWN	-2.89	1.52E-07	9.23E-06
HK2	hexokinase 2	UP	2.25	2.57E-05	4.00E-04
IDH2	isocitrate dehydrogenase 2 (NADP+), mitochondrial	DOWN	-2.26	5.00E-04	3.50E-03
IDH3A	isocitrate dehydrogenase 3 (NAD+) alpha	DOWN	-2.20	3.44E-06	9.89E-05
IDH3B	isocitrate dehydrogenase 3 (NAD+) beta	DOWN	-2.53	6.93E-05	9.00E-04
IGF1	insulin-like growth factor 1 receptor	DOWN	-2.20	3.20E-03	1.22E-02
IGFBP5	insulin like growth factor binding protein 5	DOWN	-12.84	2.06E-06	6.70E-05
IGFBP6	insulin like growth factor binding protein 6	DOWN	-2.28	5.61E-07	2.48E-05
IGFBP7	insulin like growth factor binding protein 7	DOWN	-20.02	2.26E-10	1.14E-07
IMPA2	inositol(myo)-1(or 4)-monophosphatase 2	DOWN	-2.62	5.41E-06	1.00E-04
INPP4B	inositol polyphosphate-4-phosphatase type II B	UP	2.11	6.00E-03	1.90E-02
IP6K2	inositol hexakisphosphate kinase 2	UP	2.37	2.33E-02	5.14E-

					02
ITM2B	integral membrane protein 2B	DOWN	-7.06	6.36E-07	2.74E-05
ITPK1	inositol-tetrakisphosphate 1-kinase	DOWN	-2.49	1.74E-06	5.93E-05
ITPKB	inositol-trisphosphate 3-kinase B	DOWN	-5.52	1.79E-06	6.05E-05
ITPR1	inositol 1,4,5-trisphosphate receptor, type 1	DOWN	-5.27	4.35E-05	6.00E-04
ITPR3	inositol 1,4,5-trisphosphate receptor, type 3	UP	2.31	3.00E-04	2.60E-03
ITPRIPL2	inositol 1,4,5-trisphosphate receptor interacting protein-like 2	DOWN	-2.01	1.30E-03	6.50E-03
KDSR	3-ketodihydrosphingosine reductase	DOWN	-13.58	7.27E-09	1.00E-06
LAMB2	laminin, beta 2 (laminin S)	DOWN	-2.83	1.22E-06	4.54E-05
LAMC1	laminin, gamma 1 (formerly LAMB2)	DOWN	-2.39	1.15E-05	2.00E-04
LGALS1	lectin, galactoside-binding, soluble, 1	DOWN	-20.98	1.03E-07	6.85E-06
LMCD1	LIM andcysteine-richdomains 1	DOWN	-3.98	1.50E-08	1.62E-06
LTBP1	latent transforming growth factor beta binding protein 1	DOWN	-8.63	1.89E-09	4.02E-07
MAOA	monoamineoxidase A	DOWN	-3.26	2.94E-06	8.79E-05
MAOB	monoamineoxidase B	DOWN	-9.60	4.75E-10	1.73E-07
MDH1	malate dehydrogenase 1	DOWN	-2.44	1.11E-07	7.24E-06
MDH2	malate dehydrogenase 2	DOWN	-2.42	2.12E-07	1.18E-05
MFGE8	milk fat globule-EGF factor 8 protein	DOWN	-2.07	2.68E-05	5.00E-04
MGST1	microsomal glutathione S-transferase 1	UP	5.90	2.50E-03	1.01E-02
MGST3	microsomal glutathione S-transferase 3	DOWN	-2.46	1.78E-06	6.03E-05
MIA3	melanoma inhibitory activity family, member 3	DOWN	-2.29	3.87E-07	1.86E-05
MMP2	matrix metallopeptidase 2	DOWN	-3.14	2.00E-04	1.50E-03
MPC1	mitochondrial pyruvate carrier 1	DOWN	-3.19	4.94E-11	5.64E-08
MPC2	mitochondrial pyruvate carrier 2	DOWN	-2.08	5.12E-07	2.31E-05
MRPL11	mitochondrial ribosomal protein L11	DOWN	-5.02	2.31E-05	4.00E-04
MRPL24	mitochondrial ribosomal protein L24	DOWN	-2.52	9.58E-09	1.21E-06
MRPL3	mitochondrial ribosomal protein L3	DOWN	-2.60	8.28E-05	1.00E-

					03
MRPL35	mitochondrial ribosomal protein L35	DOWN	-3.08	2.85E-05	5.00E-04
MRPL42	mitochondrial ribosomal protein L42	DOWN	-2.56	4.97E-05	7.00E-04
MRPS14	mitochondrial ribosomal protein S14	DOWN	-6.50	4.00E-10	1.59E-07
MRPS18A	mitochondrial ribosomal protein S18A	DOWN	-2.40	2.00E-04	2.00E-03
MRPS18C	mitochondrial ribosomal protein S18C	DOWN	-2.20	6.90E-06	2.00E-04
MRPS21	mitochondrial ribosomal protein S21	DOWN	-3.18	1.95E-05	4.00E-04
MRPS36	mitochondrial ribosomal protein S36	DOWN	-2.07	2.81E-05	5.00E-04
MRPS7	mitochondrial ribosomal protein S7	DOWN	-3.55	5.58E-06	1.00E-04
MSRB1	methioninesulfoxide reductase B1	DOWN	-2.42	1.34E-07	8.40E-06
MSRB2	methioninesulfoxide reductase B2	DOWN	-4.80	3.39E-10	1.50E-07
MSRB3	methioninesulfoxide reductase B3	DOWN	-5.39	6.34E-08	4.69E-06
MTR	5-methyltetrahydrofolate-homocysteine methyltransferase	DOWN	-2.39	3.00E-04	2.70E-03
MUT	methylmalonyl-CoAmutase	DOWN	-2.30	4.24E-06	1.00E-04
NDUFA1	NADH dehydrogenase (ubiquinone) 1 alpha subcomplex, 1, 7.5kDa	DOWN	2.38	2.69E-02	5.73E-02
NDUFA11	NADH dehydrogenase (ubiquinone) 1 alpha subcomplex, 11, 14.7kDa	DOWN	-6.23	1.32E-10	8.68E-08
NDUFA12	NADH dehydrogenase (ubiquinone) 1 alpha subcomplex, 12	DOWN	-5.33	3.40E-09	5.85E-07
NDUFA2	NADH dehydrogenase (ubiquinone) 1 alpha subcomplex, 2, 8kDa	DOWN	-5.29	6.40E-06	2.00E-04
NDUFA4	NDUFA4, mitochondrial complex associated	DOWN	-18.25	1.07E-08	1.30E-06
NDUFA6	NADH dehydrogenase (ubiquinone) 1 alpha subcomplex, 6, 14kDa	DOWN	-7.61	7.74E-11	6.49E-08
NDUFA9	NADH dehydrogenase (ubiquinone) 1 alpha subcomplex, 9, 39kDa	DOWN	-2.03	2.00E-04	1.80E-03
NDUFAB1	NADH dehydrogenase (ubiquinone) 1, alpha/beta subcomplex, 1, 8kDa	DOWN	-9.44	1.48E-07	9.05E-06
NDUFAF1	NADH dehydrogenase (ubiquinone) complex I, assembly factor 1	DOWN	-2.07	4.11E-07	1.95E-05
NDUFAF2	NADH dehydrogenase (ubiquinone) complex I, assembly factor 2	DOWN	-10.19	6.06E-11	5.92E-08
NDUFAF2	NADH dehydrogenase (ubiquinone) complex I, assembly factor 2	DOWN	-4.36	1.39E-08	1.55E-06
NDUFAF4	NADH dehydrogenase (ubiquinone) complex I, assembly factor 4	DOWN	-2.40	1.47E-05	3.00E-04
NDUFB10	NADH dehydrogenase (ubiquinone) 1 beta	DOWN	-3.46	2.16E-08	2.08E-

	subcomplex, 10, 22kDa				06
NDUFB3	NADH dehydrogenase (ubiquinone) 1 beta subcomplex, 3, 12kDa	DOWN	-2.81	2.93E-06	8.77E-05
NDUFB5	NADH dehydrogenase (ubiquinone) 1 beta subcomplex, 5, 16kDa	DOWN	-3.40	2.38E-06	7.47E-05
NDUFB6	NADH dehydrogenase (ubiquinone) 1 beta subcomplex, 6, 17kDa	DOWN	-5.17	4.87E-07	2.22E-05
NDUFB7	NADH dehydrogenase (ubiquinone) 1 beta subcomplex, 7, 18kDa	DOWN	-4.61	2.20E-06	7.03E-05
NDUFB9	NADH dehydrogenase (ubiquinone) 1 beta subcomplex, 9, 22kDa	DOWN	-2.73	1.98E-06	6.53E-05
NDUFS3	NADH dehydrogenase (ubiquinone) Fe-S protein 3, 30kDa (NADH-coenzyme Q reductase)	DOWN	-2.35	5.04E-07	2.28E-05
NDUFS4	NADH dehydrogenase (ubiquinone) Fe-S protein 4, 18kDa (NADH-coenzyme Q reductase)	DOWN	-3.95	8.79E-08	6.03E-06
NDUFS5	NADH dehydrogenase (ubiquinone) Fe-S protein 5, 15kDa (NADH-coenzyme Q reductase)	DOWN	-12.64	4.16E-09	6.78E-07
NDUFV2	NADH dehydrogenase (ubiquinone) flavoprotein 2, 24kDa	DOWN	-2.15	2.13E-06	6.88E-05
NEDD8	neural precursor cell expressed, developmentally down-regulated 8	DOWN	-2.05	2.71E-06	8.26E-05
NME4	NME/NM23 nucleosidediphosphate kinase 4	DOWN	-2.09	5.97E-09	8.79E-07
OAT	ornithineaminotransferase	DOWN	-2.47	2.92E-05	5.00E-04
OAZ1	ornithinedecarboxylase antizyme 1	DOWN	-3.37	2.05E-06	6.68E-05
ODC1	ornithinedecarboxylase 1	DOWN	-2.21	1.50E-03	7.20E-03
OGDH	oxoglutarate (alpha-ketoglutarate) dehydrogenase (lipoamide)	DOWN	-2.97	1.99E-07	1.13E-05
OXCT1	3-oxoacid CoA-transferase 1	DOWN	-2.22	8.00E-04	4.90E-03
PAM	peptidylglycine alpha-amidatingmonooxygenase	DOWN	-4.59	1.22E-06	4.54E-05
PCMT1	protein-L-isoaspartate (D-aspartate) O-methyltransferase	DOWN	-2.51	2.46E-07	1.32E-05
PDE10A	phosphodiesterase 10A	UP	2.38	1.86E-02	4.34E-02
PDE1C	phosphodiesterase 1C, calmodulin-dependent 70kDa	DOWN	-5.91	1.24E-06	4.58E-05
PDGFC	platelet derived growth factor C	DOWN	-2.13	1.64E-05	3.00E-04
PDHA1	pyruvate dehydrogenase (lipoamide) alpha 1	DOWN	-2.52	2.47E-08	2.27E-06
PDHB	pyruvate dehydrogenase (lipoamide) beta	DOWN	-3.46	4.74E-08	3.71E-06
PDHX	pyruvate dehydrogenase complex, component X	DOWN	-2.13	4.69E-08	3.70E-06

PDK4	pyruvate dehydrogenase kinase, isozyme 4	DOWN	-11.76	9.57E-07	3.75E-05
PELO	pelotahomolog (Drosophila); integrin alpha 1	DOWN	-2.50	2.41E-07	1.30E-05
PFAS	phosphoribosylformylglycinamidine synthase	UP	2.09	1.01E-05	2.00E-04
PGAM1	phosphoglyceratemutase 1 (brain)	DOWN	-3.62	1.53E-06	5.36E-05
PGM2L1	phosphoglucomutase 2-like 1	DOWN	-2.73	4.74E-05	7.00E-04
PGM5	phosphoglucomutase 5	DOWN	-28.42	1.63E-09	3.69E-07
PHKB	Phosphorylase kinase, beta	DOWN	-2.77	1.63E-06	5.65E-05
PICALM	phosphatidylinositol binding clathrin assembly protein	DOWN	-2.63	5.67E-07	2.50E-05
PIGP	phosphatidylinositolglycananchorbio synthesis class P	DOWN	-4.09	3.61E-07	1.76E-05
PIGT	phosphatidylinositolglycananchorbio synthesisclass T	DOWN	-2.26	8.46E-05	1.00E-03
PIGY; PYURF	phosphatidylinositolglycananchorbio synthesisclass Y; PIGY upstream reading frame	DOWN	-2.10	4.06E-06	1.00E-04
PIK3C2A	phosphatidylinositol-4-phosphate 3-kinase, catalyticsubunittype 2 alpha	DOWN	-2.32	3.07E-06	9.07E-05
PIK3CA	phosphatidylinositol-4,5-bisphosphate 3-kinase, catalytic subunit alpha	DOWN	-4.42	1.35E-08	1.51E-06
PIP4K2A	phosphatidylinositol-5-phosphate 4-kinase, type II, alpha	DOWN	-4.14	2.12E-11	4.33E-08
PITPNC1	Phosphatidylinositol transfer protein, cytoplasmic 1	DOWN	-2.44	8.00E-03	2.33E-02
PLA2G12A	phospholipase A2, group XIIA	DOWN	-2.08	2.21E-05	4.00E-04
PLA2G16	phospholipase A2, group XVI	DOWN	-2.08	7.30E-03	2.18E-02
PLA2G2F	phospholipase A2, group IIF	UP	2.18	1.47E-02	3.62E-02
PLA2G4A	phospholipase A2, group IVA (cytosolic, calcium-dependent)	DOWN	-3.23	2.11E-07	1.17E-05
PLB1	phospholipase B1	UP	2.29	8.10E-03	2.34E-02
PLCB4	phospholipase C, beta 4	DOWN	-7.50	7.80E-09	1.06E-06
PLCD4	phospholipase C, delta 4	DOWN	-2.42	8.40E-05	1.00E-03
PLCL1	phospholipase C-like 1	DOWN	-2.23	6.32E-06	2.00E-04
PLOD2	procollagen-lysine, 2-oxoglutarate 5-dioxygenase 2	DOWN	-2.11	2.64E-02	5.65E-02
PLPP1	phospholipidphosphatase 1	DOWN	-3.81	5.14E-05	7.00E-04
PLPP3	phospholipidphosphatase 3	DOWN	-4.09	4.72E-07	2.17E-

					05
PLSCR4	phospholipidscramblase 4	DOWN	-4.43	1.00E-08	1.24E-06
PMPCB	peptidase (mitochondrial processing) beta	DOWN	-3.52	1.18E-08	1.39E-06
PPA1	pyrophosphatase (inorganic) 1	DOWN	-2.77	3.00E-04	2.40E-03
PPARG	peroxisome proliferator-activated receptor gamma	UP	6.47	6.72E+00	4.50E-03
PPM1K	protein phosphatase, Mg2+/Mn2+ dependent, 1K	DOWN	-8.62	2.92E-09	5.26E-07
PPM1L	protein phosphatase, Mg2+/Mn2+ dependent, 1L	DOWN	-17.00	2.36E-09	4.60E-07
PPP2CA	protein phosphatase 2, catalytic subunit, alphaisozyme	DOWN	-2.27	2.95E-06	8.81E-05
PPP2CB	protein phosphatase 2, catalytic subunit, beta isozyme	DOWN	-3.28	6.33E-09	9.17E-07
PPP2R1A	proteinphosphatase 2, regulatory subunit A, alpha	DOWN	-3.06	5.00E-04	3.50E-03
PRKAA2	protein kinase, AMP-activated, alpha 2 catalytic subunit	DOWN	-19.76	3.28E-07	1.64E-05
PRKACB	protein kinase, cAMP-dependent, catalytic, beta	DOWN	-2.08	5.23E-07	2.35E-05
PRKCA	protein kinase C, alpha	DOWN	-5.10	2.00E-03	8.60E-03
PRKCB	protein kinase C, beta	DOWN	-2.31	8.20E-06	2.00E-04
PRPS1	phosphoribosylpyrophosphate synthetase 1	DOWN	-2.59	3.84E-08	3.21E-06
PRSS23	protease, serine, 23	DOWN	-2.46	2.00E-04	1.60E-03
PTEN	phosphatase and tensin homolog	DOWN	-2.12	7.24E-06	2.00E-04
PWP1	PWP1 homolog, endonuclein	DOWN	-2.09	2.73E-07	1.43E-05
PYGB	phosphorylase, glycogen; brain	DOWN	-33.99	1.55E-08	1.65E-06
PYGM	phosphorylase, glycogen, muscle	DOWN	-8.47	1.42E-07	8.78E-06
RARS	arginyl-tRNA synthetase	DOWN	-2.00	1.00E-04	1.40E-03
RPIA	ribose 5-phosphate isomerase A	UP	2.95	3.00E-04	2.20E-03
RPL12	ribosomal protein L12	DOWN	-3.44	1.71E-09	3.82E-07
RPL13	ribosomal protein L13	DOWN	-6.57	2.22E-10	1.14E-07
RPL13A	ribosomal protein L13a	DOWN	-22.61	1.62E-08	1.71E-06
RPL14	ribosomal protein L14	DOWN	-3.07	1.52E-07	9.20E-06
RPL15	ribosomal protein L15	DOWN	-2.05	5.24E-06	1.00E-

					04
RPL17	ribosomal protein L17	DOWN	-2.03	7.29E-06	2.00E-04
RPL18	ribosomal protein L18	DOWN	-4.18	9.53E-08	6.41E-06
RPL18A	ribosomal protein L18a	DOWN	-10.32	1.38E-08	1.54E-06
RPL19	ribosomal protein L19	DOWN	-2.53	1.25E-07	7.94E-06
RPL21	ribosomal protein L21	DOWN	-23.51	4.55E-08	3.61E-06
RPL24	ribosomal protein L24	DOWN	-4.48	1.13E-06	4.24E-05
RPL26	ribosomal protein L26	DOWN	-4.31	2.30E-07	1.26E-05
RPL27	ribosomal protein L27	DOWN	-6.93	3.60E-06	1.00E-04
RPL30	ribosomal protein L30	DOWN	-2.60	8.22E-07	3.35E-05
RPL35	ribosomal protein L35	DOWN	-2.36	1.63E-06	5.65E-05
RPL35A	ribosomal protein L35a	DOWN	-2.11	1.23E-08	1.42E-06
RPL36	ribosomal protein L36	DOWN	-2.06	2.41E-06	7.54E-05
RPL37	ribosomal protein L37	DOWN	-5.54	1.35E-05	3.00E-04
RPL38	ribosomal protein L38	DOWN	-2.40	3.78E-08	3.17E-06
RPL4	ribosomal protein L4	DOWN	-8.15	9.61E-07	3.76E-05
RPL41	ribosomal protein L41	DOWN	-2.38	2.73E-07	1.43E-05
RPL5	ribosomal protein L5	DOWN	-3.09	4.50E-11	5.43E-08
RPL6	ribosomal protein L6	DOWN	-9.13	4.72E-09	7.48E-07
RPL8	ribosomal protein L8	DOWN	-6.80	4.51E-09	7.24E-07
RPL9	ribosomal protein L9	DOWN	-2.40	4.17E-08	3.40E-06
RPLP0	ribosomalprotein, large, P0	DOWN	-2.76	2.02E-06	6.62E-05
RPLP1	ribosomalprotein, large, P1	DOWN	-3.80	5.20E-12	1.95E-08
RPS10	ribosomal protein S10	DOWN	-2.08	1.25E-06	4.61E-05
RPS11	ribosomal protein S11	DOWN	-10.30	6.91E-07	2.94E-05
RPS13	ribosomal protein S13	DOWN	-51.68	7.28E-11	6.49E-08
RPS14	ribosomal protein S14	DOWN	-2.41	1.36E-07	8.50E-

					06
RPS15	ribosomal protein S15	DOWN	-10.26	1.68E-08	1.76E-06
RPS15A	ribosomal protein S15a	DOWN	-5.23	8.35E-10	2.49E-07
RPS16	ribosomal protein S16	DOWN	-25.84	3.33E-08	2.85E-06
RPS18	ribosomal protein S18	DOWN	-3.28	1.34E-05	3.00E-04
RPS19	ribosomal protein S19	DOWN	-7.24	6.01E-09	8.82E-07
RPS2	ribosomal protein S2	DOWN	-4.29	1.08E-10	7.95E-08
RPS20	ribosomal protein S20	DOWN	-2.58	2.99E-07	1.53E-05
RPS21	ribosomal protein S21	DOWN	-3.32	1.08E-06	4.10E-05
RPS23	ribosomal protein S23	DOWN	-2.94	4.00E-10	1.59E-07
RPS25	ribosomal protein S25	DOWN	-5.52	1.95E-07	1.12E-05
RPS26	ribosomal protein S26	DOWN	-2.48	2.35E-05	4.00E-04
RPS27	ribosomal protein S27	DOWN	-2.14	9.87E-09	1.23E-06
RPS28	ribosomal protein S28	DOWN	-2.14	7.60E-08	5.37E-06
RPS29	ribosomal protein S29	DOWN	-2.50	2.43E-07	1.31E-05
RPS3	ribosomal protein S3	DOWN	-3.68	9.09E-09	1.16E-06
RPS3A	ribosomal protein S3a	DOWN	-4.37	6.14E-10	2.02E-07
RPS4X	ribosomal protein S4, X-linked	DOWN	-3.90	2.04E-08	2.00E-06
RPS5	ribosomal protein S5	DOWN	-12.72	5.37E-09	8.21E-07
RPS9	ribosomal protein S9	DOWN	-34.45	1.10E-09	2.91E-07
RSL1D1	ribosomal L1 domain containing 1	DOWN	-2.35	3.91E-07	1.87E-05
RSL24D1	ribosomal L24 domain containing 1	DOWN	-4.60	2.77E-08	2.50E-06
S1PR3	sphingosine-1-phosphate receptor 3	DOWN	-2.73	9.14E-07	3.64E-05
SARS	seryl-tRNA synthetase	DOWN	-3.05	8.97E-08	6.12E-06
SAT2	spermidine/spermine N1-acetyltransferase familymember 2	DOWN	-2.43	2.68E-07	1.41E-05
SDC2	syndecan 2	DOWN	-3.34	1.70E-09	3.82E-07
SDHC	succinate dehydrogenase complex, subunit C,	DOWN	-2.31	2.10E-03	9.00E-

	integral membrane protein, 15kDa				03
SDHD	succinate dehydrogenase complex subunit D, integral membrane protein	DOWN	-5.21	7.62E-08	5.38E-06
SDPR	serum deprivation response	DOWN	-13.78	7.14E-09	9.94E-07
SERINC1	serine incorporator 1	DOWN	-3.61	2.78E-09	5.18E-07
SIAH1	siah E3 ubiquitin protein ligase 1	DOWN	-3.23	2.00E-04	2.00E-03
SLC16A7	solute carrier family 16 (monocarboxylate transporter), member 7	DOWN	-2.01	1.93E-05	4.00E-04
SLC25A5	solute carrier family 25 (mitochondrial carrier; adeninenucleotide translocator), member 5	DOWN	-2.62	2.67E-06	8.16E-05
SLC25A6	solute carrier family 25 (mitochondrial carrier; adeninenucleotide translocator), member 6	DOWN	-2.08	3.76E-06	1.00E-04
SLC2A1	solute carrier family 2 (facilitated glucose transporter), member 1	UP	2.66	4.30E-03	1.49E-02
SLC2A12	solute carrier family 2 (facilitated glucose transporter), member 12	DOWN	-2.96	1.42E-05	3.00E-04
SLC2A3	solute carrier family 2 (facilitated glucose transporter), member 3	DOWN	-3.22	1.21E-06	4.51E-05
SLC2A4	solute carrier family 2 (facilitated glucose transporter), member 4	DOWN	-2.13	9.12E-06	2.00E-04
SLC35A1	solute carrier family 35 (CMP-sialicacid transporter), member A1	DOWN	-3.55	5.55E-07	2.46E-05
SMPD2	sphingomyelinphosphodiesterase 2, neutral membrane (neutral sphingomyelinase)	UP	2.29	7.68E-05	1.00E-03
SMS	spermine synthase	DOWN	-2.57	1.04E-05	2.00E-04
SOD1	superoxide dismutase 1, soluble	DOWN	-40.66	8.52E-11	6.77E-08
SPARCL1	SPARC like 1	DOWN	-127.65	1.30E-08	1.48E-06
SPCS2	signalpeptidase complex subunit 2	DOWN	-3.68	1.85E-05	3.00E-04
SPCS3	signalpeptidase complex subunit 3	DOWN	-2.32	1.00E-04	1.20E-03
SPPL2A	signal peptidepeptidase like 2A	DOWN	-3.20	3.97E-05	6.00E-04
SUCLG1	succinate-CoAligase, alpha subunit	DOWN	-3.09	2.42E-08	2.24E-06
TALDO1	transaldolase 1	DOWN	-5.41	2.09E-08	2.04E-06
TMEM208	transmembrane protein 208	DOWN	-3.65	6.90E-07	2.94E-05
TNC	tenascin C	DOWN	-2.33	3.10E-02	6.39E-02
TXN	thioredoxin	DOWN	-13.15	1.96E-07	1.12E-05
TXNRD1	thioredoxin reductase 1	DOWN	-2.77	1.88E-06	6.29E-

					05
UBA52	ubiquitin A-52 residue ribosomal prot. fusion product 1	DOWN	-5.37	2.23E-07	1.23E-05
UBB	ubiquitin B	DOWN	-2.68	5.99E-11	5.92E-08
UBE2L3	ubiquitin conjugating enzyme E2L 3	DOWN	-2.48	1.26E-05	3.00E-04
UBE2M	ubiquitin-conjugating enzyme E2M	DOWN	-3.21	2.00E-04	1.60E-03
UBL5	ubiquitin-like 5	DOWN	-7.60	1.46E-08	1.59E-06
UGDH	UDP-glucose 6-dehydrogenase	DOWN	-2.14	5.94E-07	2.60E-05
UGP2	UDP-glucosepyrophosphorylase 2	DOWN	-2.57	2.23E-09	4.45E-07
UQCR10	ubiquinol-cytochrome c reductase, complex III subunit X	DOWN	-2.30	1.17E-02	3.06E-02
UQCR11	ubiquinol-cytochrome c reductase, complex III subunit XI	DOWN	-2.85	8.56E-07	3.46E-05
UQCRB	ubiquinol-cytochrome c reductase binding protein	DOWN	-2.80	3.01E-10	1.38E-07
UQCRC2	ubiquinol-cytochrome c reductase coreprotein II	DOWN	-2.82	9.14E-07	3.64E-05
UQCRFS1	ubiquinol-cytochrome c reductase, Rieskeiron-sulfur polypeptide 1	DOWN	-3.45	6.00E-07	2.62E-05
UQCRH	ubiquinol-cytochrome c reductase hinge protein	DOWN	-2.48	1.54E-05	3.00E-04
UQCRHL	ubiquinol-cytochrome c reductase hinge protein like	DOWN	-3.20	2.00E-04	2.00E-03
USP25	ubiquitin specific peptidase 25	DOWN	-2.18	3.07E-05	5.00E-04
USP9X	ubiquitin specific peptidase 9, X-linked	DOWN	-2.42	7.18E-08	5.13E-06
WFS1	Wolfram syndrome 1 (wolframin)	DOWN	-3.02	7.59E-07	3.15E-05
YME1L1	YME1-like 1 ATPase	DOWN	-2.56	1.64E-07	9.76E-06
YWHAB	tyrosine 3-monooxygenase/tryptophan 5-monooxygenase activation protein, beta	DOWN	-2.58	1.00E-04	1.20E-03
YWHAE	tyrosine 3-monooxygenase/tryptophan 5-monooxygenase activationprotein, epsilon	DOWN	-3.71	8.10E-06	2.00E-04

NOTE: FC= Fold Change; FDR p-val= FDR p-value.

Table S5. Putative binding motif enrichment analysis using the Enrich webtool and showing the relative relevance of various transcription factors in the deregulated transcripts.

Term	p-value	Adj. *p*-value	Genes
**E2F1**	6.3E-22	4.0E-19	RPL4,RPL30,ACAA2,NDUFA12,ATP5C1,ADARB1,RPL8,PWP1,RPL6,UBE2L3,RPS15,RPS14,RPS16,RPL18A,RPS18,PPP2R1A,PMPCB,RPL35,RPL37,RPS11,ATP6V1E1,RPS10,PELO,B2M,PDHX,RPS9,RPL21,FNBP1,RPS5,APLP2,SARS,MRPS18A,SDHC,EEF1A1,SMS,RPL24,RPL27,SUCLG1,RPL26,UQCRC2,UQCRB,NDUFB10,COX15,MAOA,GMPS,AK2,I

			TPR1,COX7A2,ATP5O,OAZ1,UQCRH,MRPL11,ATP5L,ATP5B,ATP5E,ATP5D,COX11,IGFBP7,NDUFV2,ATP6V1F,DARS,RPL41,PDHA1,IDH2,GOT2,H3F3A,FN1,MRPL24,RPS28,RPS27,CALU,OGDH,RPS20,SERINC1,CALM1,RPS21,CALM2,ITM2B,RPS23,FARSB,PICALM,YWHAE,COX7B,MRPS14,RPLP1,PIGP,PTEN,SLC2A1,MTR,SLC2A3,LAMC1,ATP5G3,ATP5G2,COX7C,MRPL35,PITPNC1,CYR61,HK2,PIGY,MRPL3,ATIC,CSRP2,ALDH2,RARS,ACADM,DLAT,IDH3A,COX8A,ATP6AP1,ITPK1,RPL13A,RPS3A,NME4,ATP5F1,EEF1G,HADHB,PLCB4,CAT,NDUFS3,SLC25A5,ALDH9A1,PRPS1,OAT,NDUFB7,MRPS36,RPL12,ATP5A1,ODC1,NDUFB3,COX5B,COX5A,FSTL1,PPP2CA,CST3,PPP2CB,RPS15A,RPS3,CKB,RPL15,RPS2,RPL18,RPL17,RPL19,BCHE,NDUFA6,MDH1,MDH2,USP9X,NDUFA4,NDUFA2,NDUFA1,COX6C,ASS1,SOD1,SPCS3,RSL1D1,SPCS2,MSRB2
**EKLF**	2.9E-20	9.3E-18	RPL4,HIBADH,MRPS14,NDUFA12,RPLP1,SLC2A1,SLC2A4,ATP5G2,PFAS,PWP1,RPL6,RPS4X,MRPL3,RPS14,ATIC,LGALS1,RPS16,ALDH2,RPS18,IDH3B,PMPCB,RPL35,RPS11,COX8A,RPS9,GSTO1,ECH1,SARS,RPL13A,SDHC,NME4,EEF1A1,EEF1G,CAT,RPL24,RPL27,SUCLG1,SLC25A5,ALDH9A1,OAT,UQCRB,NDUFB10,MGST3,NDUFB3,GMPS,ATP5J,NEDD8,ATP5O,MRPL11,ACAT1,PPP2CA,CST3,OXCT1,RPS3,EIF4EBP2,RPL15,RPL19,MDH1,MDH2,NDUFA4,TXNRD1,GOT2,H3F3A,IDH2,RPIA,RPS26,RPS25,RSL1D1,RPS28,SPCS2,NDUFAB1,CALM3,ITM2B,UBE2M,RPS23
**MYC**	7.8E-20	1.7E-17	YWHAE,RPL4,RPL5,COX7B,RPL30,RPLP1,ATP5C1,RPL8,ATP5G2,COX7C,PWP1,PIGY,RPL6,GLS,RPS15,MRPL3,RPS14,ATIC,RPS19,RPL18A,RPS18,RARS,RPL36,RPL35,RPL38,RPL37,RPS13,COX8A,RPS9,RPL21,RPS5,SARS,NME4,RPS3A,ATP5F1,EEF1A1,EEF1G,NDUFS4,NDUFS3,YME1L1,RPL27,RPL26,NDUFB5,RPL12,ATP5A1,ODC1,MRPL11,ATP5B,RPS15A,ATP5E,RPS3,EIF4EBP2,RPL13,RPL15,RPS2,RPL18,RPL17,COX10,ATP6V1F,DARS,NDUFA4,GOT2,H3F3A,MRPL24,SOD1,RPS25,RSL1D1,RPS27,RPS29,RPS20,RPS21,FARSB,RPS23
**ESRRB**	2.5E-18	3.1E-16	HIBADH,YWHAB,NDUFA12,COX4I1,ATP5C1,PYGM,ATP5G3,COX6A1,ATP5G2,COX7C,PIGY,GLS,RPS15,CSRP2,AUH,IDH3B,UQCRFS1,DLAT,AASS,IDH3A,COX8A,PLA2G12A,GPX4,PGAM1,APLP2,SDHC,SDHD,NDUFS5,NDUFS4,NDUFS3,SUCLG1,UQCRC2,GAS6,DLD,NDUFB9,PRKAA2,UQCRB,NDUFB10,NDUFB5,MRPS36,MGST3,ATP5A1,ODC1,GMPS,AK2,COX7A2,PPM1K,PDHB,ATP5O,COX5B,COX5A,COX7A1,LTBP1,UQCRH,ACAT1,ATP5B,OXCT1,ATP5E,PDGFC,ATP5D,COX11,CKB,NDUFV2,COX10,NDUFA9,NDUFA6,MDH1,MDH2,NDUFA2,COX6C,NDUFAB1,OGDH,ACO1,CALM3,CHPT1,CALM2,ITM2B,UBE2M
**XRN2**	8.1E-18	8.5E-16	YWHAE,RPL4,RPL5,NDUFA11,MRPS14,RPLP0,AMD1,RPL8,MRPL35,RPL9,RPL6,UBE2L3,RPS4X,RPS14,RPS19,RPS18,RPL36,IDH3B,RPL35,RPL38,RPL37,B2M,RPS10,RPS9,RPS5,SARS,MRPS21,RPL13A,SDHC,MRPS7,EEF1A1,NDUFS5,NDUFS3,RPL24,RPL27,RPL26,GAPDH,FH,OAT,NDUFB

			6,RPL12,ITPR1,ATP5J,TXN,OAZ1,ACAT1,ATP5B,UGP2,RPS15A,UBB,RPL14,COX11,RPL13,RPL15,CKB,RPS2,RPL18,RPL17,IP6K2,RPL19,RPL41,MDH1,NDUFA2,RPL35A,MRPL24,SOD1,RPS26,RPS28,RPS27,GSTA4,RPS29,CYCS,CALM3,SERINC1,RPS20,CALM1,CALM2,RPS21,UBE2M,FARSB
**NELFA**	3.5E-13	2.5E-11	YWHAE,PYGB,ACAA2,HIBADH,NDUFA12,AMD1,PTEN,SLC2A1,ATP5C1,MTR,SLC2A3,ATP5G3,PIK3C2A,RPL8,RPL6,GLS,RPS15,PCMT1,CSRP2,ALDH2,RPS18,IMPA2,ACADM,RPL37,DLAT,RPS11,RPS13,PDHX,RPS9,RPL21,GPX4,FNBP1,APLP2,RPL13A,SDHC,NME4,SDHD,CIT,EEF1A1,ITPKB,UGDH,PPA1,CAT,UQCRC2,RPL26,GAPDH,UQCRB,NDUFB6,RPL12,ATP5A1,GMPS,ITPR1,ACACB,ADH5,PPP2CA,ATP5B,UGP2,UBB,RPS3,EIF4EBP2,RPL13,CKB,RPS2,ATP6V0A1,RPL19,SLC35A1,DARS,RPL41,NDUFA6,NDUFA4,H3F3A,FN1,SOD1,RPS26,RSL1D1,RPS28,SPCS2,RPS27,CALU,OGDH,RPS20,CALM1,RPS21,CALM2
**TTF2**	5.8E-13	3.7E-11	YWHAE,RPL4,RPL5,MRPS14,RPLP1,RPLP0,AMD1,COX7C,RPL9,RPL6,RPS15,RPS4X,MRPL3,RPS14,RPS18,RPL36,RPL35,RPL38,RPL37,RPS11,PRKACB,RPS13,RPS9,RPS3A,ACSL3,MRPS7,EEF1A1,EEF1G,NDUFS4,NDUFS3,RPL24,UQCRC2,RPL26,GAPDH,UQCRB,NDUFB10,RPL12,MGST3,ITPR1,COX7A2,PDHB,RPS15A,UBB,RPL14,RPS3,RPL13,RPL15,RPS2,RPL18,RPL17,RPL19,RPL41,NDUFA4,H3F3A,MRPL24,SOD1,RSL1D1,RPS28,RPS27,NDUFAF4,CYCS,CALM3,CHPT1,SERINC1,RPS20,CALM1,CALM2,PAM,UBE2M,RPS23
**ETS1**	1.2E-12	7.1E-11	RPL5,COX7B,COX4I1,RPLP0,MTR,RPL8,COX7C,RPL9,RPL6,RPS15,UBL5,RPS14,MRPL42,MRPL3,RPS16,RPS19,RARS,IDH3B,RPL36,UQCRFS1,RPL38,RPL37,ATP6V1E1,RPS11,RPS13,RPS9,RPL21,RPS5,RPL13A,RPS3A,SDHD,PGM2L1,MRPS18C,RPL24,YME1L1,SUCLG1,RPL27,UQCRC2,RPL26,UBA52,NDUFB7,NDUFB10,RPL12,MGST3,NDUFB3,ITPR1,COX7A2,ATP5O,MRPL11,RPS15A,ATP5D,RPL14,RPS3,TMEM208,RPL13,ATP6V1F,NDUFA9,SLC35A1,NDUFA4,PHKB,MRPL24,RPS26,RPIA,RPS25,RPS27,NDUFAB1,CYCS,RPS20,CALM1,RPS21,FARSB,RPS23
**ZFX**	8.6E-11	4.6E-09	RPL30,NDUFA12,ATP5C1,ADARB1,RPL8,PWP1,RPL6,UBE2L3,RPS14,PCMT1,RPS16,RPS19,RPL18A,RPS18,PPP2R1A,RPL36,RPL37,RPS11,PDHX,FNBP1,RPS5,MRPS18A,SDHD,EEF1A1,RPL24,RPL27,RPL26,UQCRC2,UBA52,NDUFB10,GMPS,AK2,OAZ1,LTBP1,COX7A1,ADH5,ACAT1,MRPL11,ATP5L,ATP5B,UBB,ATP5E,ESD,ATP5D,ABL1,NDUFV2,DARS,RPS27,RPS29,OGDH,RPS20,ACO1,CALM1,RPS21,ITM2B,RPS23,UBE2M,HIBADH,YWHAB,RPLP1,PIGP,PTEN,MTR,ATP5G2,COX7C,MRPL3,ATIC,AUH,IMPA2,RARS,UQCRFS1,DLAT,IDH3A,COX8A,GSTO1,ITPK1,NME4,UGDH,NDUFS3,SLC25A5,DLD,RPL12,ATP5A1,PDHB,COX5B,COX5A,PPP2CA,PPP2CB,RPS15A,RPL14,RPS3,RPL13,RPL15,RPS2,RPL18,SLC35A1,NDUFA6,MDH1,LAMB2,USP9X,NDUFA4,TXNRD1,COX6C,SOD1,RPIA,RSL1D1,GNPDA2,NDUFAB1,MSRB2
**VDR**	1.5E-09	7.1E-08	YWHAE,RPL5,COX7B,NDUFA11,MRPS14,NDUFA12,AM

			D1,RPLP0,PTEN,SLC2A3,MRPL35,PFAS,UBE2L3,RPS15,RPS14,MRPL3,RPS19,AUH,RPL36,PMPCB,RPL38,RPL37,RPS11,RPS9,GSTO2,RPS5,ECH1,MRPS21,RPL13A,RPS3A,MRPS18C,HADHB,RPL24,YME1L1,SUCLG1,RPL27,UQCRC2,RPL26,UBA52,NDUFB7,NDUFB6,NDUFB5,ODC1,NDUFB3,ATP5I,NEDD8,COX7A2,PPM1K,COX5B,ACAT1,ATP5L,PPP2CA,UBB,OXCT1,ATP5E,TMEM208,RPL13,RPL19,ATP6V1F,NDUFA9,RPL41,NDUFA6,MDH1,RPL35A,COX6C,MRPL24,RPS26,RPIA,SPCS3,RPS27,NDUFAB1,CALU,CYCS,RPS20,CALM3,NDUFAF1,RSL24D1,RPS21,CALM2,FARSB,RPS23
**HOXC9**	2.3E-09	1.1E-07	COX7B,RPL30,PIGT,HIBADH,MRPS14,NDUFA12,RPLP1,SLC2A3,ATP5G3,COX7C,MRPL35,PIGY,RPS15,UBL5,MRPL3,ATIC,LGALS1,RPS16,RPS19,IDH3B,RPL38,RPL37,ATP6V1E1,B2M,RPS9,APLP2,MRPS21,MRPS18A,RPS3A,SDHD,ACSL3,MUT,COX6B1,HADHA,CAT,NDUFS4,RPL24,YME1L1,UQCRC2,RPL26,MFGE8,DLD,OAT,NDUFB7,MRPS36,NDUFB5,ITPR1,ATP5I,PPM1K,COX5B,UQCRH,RPS15A,UBB,ATP5E,ATP5D,ESD,TMEM208,RPL15,RPS2,RPL17,IP6K2,NDUFA9,NDUFA6,MDH2,COX6C,RPS26,RPS25,RSL1D1,RPS28,RPS29,NDUFAF4,NDUFAB1,NDUFAF2,CALU,NDUFAF1
**SPI1**	1.1E-07	4.7E-06	YWHAE,RPL30,PIGT,ACAA2,MRPS14,YWHAB,ATP5C1,SLC2A3,ATP5G3,COX6A1,RPL8,PITPNC1,RPL6,GLS,RPS15,RPS14,ATIC,RPS16,RPS19,RPL18A,IMPA2,RARS,IDH3B,RPL35,RPL38,RPS11,IDH3A,GSTO2,RPL21,PGAM1,RPL13A,PLA2G4A,MRPS18A,RPS3A,EEF1G,ITPKB,NDUFS4,SUCLG1,RPL27,GAPDH,NDUFB9,NDUFB5,AK2,ITPR1,NEDD8,PDHB,COX5B,COX7A1,ACAT1,PPP2CA,CST3,ITPRIPL2,RPL14,RPS3,EIF4EBP2,RPL13,RPL17,COX10,NDUFA9,DARS,ATP8B2,GOT2,RPS26,RPS27,SERINC1, RSL24D1,RPS21,CALM2,UBE2M
**YY1**	3.7E-07	1.5E-05	YWHAE,RPL4,APP,COX7B,RPL30,MRPS14,AMD1,LAMC1,ATP5G2,MRPL35,COX7C,UBE2L3,MRPL42,RPL36,IDH3B,RPS11,COX8A,PDHX,ECH1,RPL13A,MRPS18A,SDHC,MRPS7,MRPS18C,EEF1A1,INPP4B,DPYD,NDUFS4,YME1L1,UBA52,DGKH,UQCRB,RPL12,ATP5A1,PDHB,OAZ1,LTBP1,UQCRH,MRPL11,ATP5L,UBB,RPS3,RPL18,COX10,ATP6V0A1,NDUFA9,NDUFA2,RPL35A,MRPL24,RPS26,RPS28,SPCS2,RPS27,OGDH, ITM2B
**CEBPB**	1.0E-06	3.9E-05	COX7B,NDUFA11,PIGP,SAT2,ATP5G3,PIK3C2A,CYR61,RPL6,RPS15,RPS4X,UBL5,RPS14,LGALS1,CSRP2,RPS19,UQCRFS1,RPL38,ATP6V1E1,RPS10,PELO,COX8A,PDHX,RPS9,GSTO2,FNBP1,ECH1,MRPS21,SDHD,DNAJC3,SUCLG1,RPL27,SLC25A5,UQCRB,ATP5A1,ODC1,ITPR1,ATP5H,UQCR10,COX5B,ATP5O,COX7A1,MRPL11,ITPRIPL2,PPP2CB,UBB,RPL14,RPL15,COX10,IP6K2,RPL19,USP25,ATP8B2,NDUFA4,WFS1,NDUFA2,H3F3A,NDUFA1,PHKB,RPS26,RPIA,RPS25,RPS27,RPS29,NDUFAF4,CYCS,RPS20,PICALM
**GABP**	1.6E-06	5.5E-05	RPL30,PIGT,NDUFB7,NDUFB5,NDUFB3,COX5B,COX6A1,COX7C,UQCRH,RPL6,MRPL3,UBL5,RPS14,RPS18,RARS,I

			DH3B,PMPCB,ATP6V1E1,COX10,COX8A,RPL41,MDH1,RPS5,MRPS21,RPL35A,MRPS18A,SDHC,RPS3A,SDHD,MRPL24,MRPS18C,RPS25, RPS27,CALU,NDUFS3,YME1L1,RPL27,SERINC1,RPS20,RPL26,UBE2M
**CREM**	2.0E-06	6.8E-05	RPL5,RPL30,ACAA2,ATP5C1,COX6A1,RPL8,PWP1,RPL6,GLS,RPS14,PCMT1,RPS16,RPS19,RPL18A,RPS18,PPP2R1A,RPL36,SLC16A7,CYP1B1,RPL38,RPS11,PRKACB,PELO,RPS13,RPS9,RPL21,IGFBP5,RPS5,APLP2,SDHC,ACSL3,EEF1A1,RPL24,RPL27,SUCLG1,RPL26,UBA52,NDUFB10,MAOA,GMPS,AK2,ATP5J,COX7A2,ATP5O,OAZ1,ADH5,ACAT1,ATP5B,UBB,ATP5D,ABL1,COX11,EIF4EBP2,NDUFV2,ATP6V1F,ATP6V0A1,DARS,RPL41,H3F3A,FN1,RPS26,RPS27,RPS29,NDUFAF4,NDUFAF2,CALU,OGDH,RPS20,CALM3,SERINC1,CALM1,CALM2,UBE2M,FARSB,PICALM,YWHAE,COX4I1,RPLP0,AMD1,PTEN,MTR,LAMC1,PIK3C2A,ATP5G2,PFAS,PRSS23,CYR61,AUH,IMPA2,RARS,IDH3B,UQCRFS1,DLAT,IDH3A,COX8A,PLA2G12A,GSTO2,GPX4,PGAM1,RPL13A,RPS3A,PGM2L1,CIT,EEF1G,DNAJC3,HADHB,ITPKB,HADHA,UGDH,NDUFS3,PPARG,GAPDH,DLD,OAT,NDUFB6,PPM1L,MRPS36,ATP5A1,ODC1,PDHB,COX5B,COX5A,PPP2CA,PPP2CB,UGP2,RPL14,RPS3,RPL13,TMEM208,CKB,RPL18,SLC35A1,USP25,NDUFA6,MDH1,MDH2,USP9X,TXNRD1,RPL35A,COX6C,CDC42BPA,SOD1,SPCS3, RSL1D1,SPCS2,PDE10A,MSRB3,CHPT1
**PPARG**	3.4E-06	1.0E-04	ACAA2,COX15,YWHAB,ECH1,MGST1,AK2,SDHC,ATP5G3,COX5A,SOD1,C3, HADHB,RPS19,SDPR,CYCS,PPARG,CHPT1,ACADM
**FLI1**	3.7E-06	1.1E-04	RPL4,CHRM3,RPL30,NDUFA12,TNC,ATP5C1,COX6A1,PWP1,PLB1,RPL6,UBE2L3,RPS15,RPS14,PCMT1,LGALS1,RPS16,RPS19,RPL18A,RPL36,RPL38,RPL37,RPS11,ATP6V1E1,RPL21,IGFBP5,FNBP1,PRKCB,RPS5,APLP2,SARS,SDHC,PRKCA,SDHD,COX6B1,MRPS18C,PLA2G16,INPP4B,SDPR,RPL24,SPARCL1,YME1L1,RPL27,RPL26,UBA52,ITPR1,ATP5J,ATP5I,ITPR3,ATP5H,ATP5O,LTBP1,UQCRH,ADH5,ATP5L,C3,EIF4EBP2,IGFBP7,NDUFV2,COX10,DARS,RPL41,GOT2,H3F3A,PHKB,IGF1,MRPL24,RPS26,RPS25,RPS27,RPS29,CALU,OGDH,CALM3,SERINC1,NDUFAF1,CALM1,RPS21,RSL24D1,RPS23,UBE2M,PICALM,YWHAE,PIGT,MRPS14,YWHAB,RPLP1,COX4I1,AMD1,PIGP,SLC2A1,MIA3,SLC2A3,ATP5G2,COX7C,UBL5,MRPL3,ATIC,IMPA2,RARS,CYP4V2,IDH3B,UQCRFS1,COX8A,GSTO2,GLRX3,GPX4,GSTO1,MRPS21,RPS3A,MUT,EEF1G,ITPKB,HADHA,UGDH,PIK3CA,NDUFS5,NDUFS3,MFGE8,GAPDH,DLD,ALDH9A1,SLC25A6,NDUFB9,NDUFB6,NDUFB5,RPL12,NDUFB3,COX5B,PPP2CA,CST3,RPS3,TMEM208,RPL15,RPL19,GSN,NDUFA6,MDH1,MDH2,PLCL1,TXNRD1,NDUFA2, CDC42BPA, ASS1,RPIA,RSL1D1,PDE10A,GNPDA2,NDUFAB1
**SOX2**	7.4E-06	2.1E-04	YWHAE,COX7B,COX4I1,AMD1,PTEN,SLC2A3,ATP5G2,COX7C,PITPNC1,CYR61,HK2,UBL5,RPS14,RPS19,ALDH2,R

			PS18,PMPCB,RPS11,IDH3A,COX8A,ENTPD1,RPL21,FNBP1,RPS5,RPL13A,SARS,SDHC,RPS3A,PGM2L1,CIT,EEF1A1,EEF1G,BACE1,UGDH,SMS,RPL27,GAPDH,PRPS1,ITPR1,PDHB,ATP5H,COX5A,OAZ1,FSTL1,COX7A1,CST3,UBB,PDGFC,ATP5D,CKB,NDUFV2,RPL18,ATP8B2,FN1,IGF1,COX6C,MRPL24,RPS26,RPIA,GSTA4,CALM1,RPS21,PLCD4,RPS23
**OCT4**	7.5E-06	2.1E-04	RPL5,SLC2A1,SLC2A3,RPL8,RPL9,CYR61,HK2,GLS,RPS4X,RPS14,RPS16,RPS19,ALDH2,RPS18,RPS11,PRKACB,ENTPD1,RPL21,FNBP1,RPS5,RPL13A,SARS,RPS3A,CIT,EEF1A1,BACE1,UGDH,PLCB4,SMS,SPARCL1,RPL27,GAPDH,PRPS1,ITPR1,NEDD8,COX7A2,COX5B,COX5A,OAZ1,COX7A1,CST3,ATP5B,UBB,PDGFC,RPS3,ABL1,CKB,RPS2,RPL18,ATP6V1F,AOC3,ATP8B2,GOT2,FN1,COX6C,ASS1,RPS26,RPIA,GSTA4,RPS20, CALM1,RPS21,UBE2M,FARSB
**CHD1**	8.5E-06	2.5E-04	YWHAE,RPL5,APP,MRPS14,SPPL2A,SLC2A1,ATP5C1,MIA3,LAMC1,ATP5G2,COX7C,MRPL35,CYR61,GLS,UBL5,CSRP2,IMPA2,CKMT1B,ACADM,RPS13,COX8A,GSTO2,GLRX3,PGAM1,SARS,MRPS18A,RPS3A,ACSL3,ITPKB,PPA1,UQCRC2,ALDH9A1,PPM1L,COX15,SDC2,MGST3,AK2,UQCR11,UQCRH,ATP5L,UBB,CKB,NDUFV2,IP6K2,USP25,ATP8B2,USP9X,LAMB2,CKMT1A,TXNRD1,GOT2,CDC42BPA,RPS26,RPS25,RPS28, RPS27,RPS29,OGDH,MSRB2,CHPT1,CALM1,RPS21,CALM2,PICALM
**BCL3**	1.0E-05	2.6E-04	YWHAE,RPL5,NDUFB10,NDUFB5,ATP5A1,PIGP,AMD1,ATP5J,PYGM,UQCR11,LAMC1,RPL8,COX5A,CYR61,HK2,MRPL11,ATP5B,PCMT1,UGP2,RPL18A,UBB,ATP5D,IDH3B,PELO,RPL17,PDHX,RPL41,RPS5,ECH1,MRPS18A,SOD1,SPCS2,CYCS,RPS21, ITM2B
**TAL1**	1.3E-05	3.1E-04	RPL4,NDUFA11,MRPS14,COX4I1,RPLP0,PTEN,SLC2A1,ATP5G3,RPL8,RPL6,RPS15,RPS14,ATIC,RPS19,PPP2R1A,RPS18,RPL36,RPS10,B2M,RPS9,GLRX3,GPX4,RPS5,MRPS7,EEF1A1,RPL27,RPL26,UBA52,ALDH9A1,NDUFB9,NDUFB10,NDUFB6,RPL12,ATP5A1,ODC1,NDUFB3,ITPR1,UQCR11,COX5A,PPP2CA,ATP5B,UBB,RPL14,RPS3,COX11,RPL13,RPL18,RPL17,RPL19,DARS,RPL41,NDUFA6,MDH2,TXNRD1,RPL35A,PHKB, RPS26,RPS25,RPS27,PDE10A,RPS20,CALM1,RPS21,UBE2M,RPS23
**RUNX2**	1.7E-05	3.9E-04	PIGT,TNC,ATP5C1,PITPNC1,CYR61,HK2,GLS,LGALS1,RPS19,RPL18A,ALDH2,CYP1B1,DLAT,RPS10,PELO,LMCD1,IGFBP5,FNBP1,GSTO1,PGAM1,MMP2,APLP2,ITPK1,MRPS18A,NME4,MRPS7,CIT,EEF1A1,EEF1G,BACE1,UGDH,PLA2G16,PLCB4,RPL26,GAS6,PRKAA2,OAT,COX15,AK2,ATP5H,UQCR10,ACACB,LTBP1,ADH5,MRPL11,UGP2,PDGFC,ATP6V0A1,RPL19,SLC35A1,DARS,GSN,MDH1,ATP8B2,LAMB2,TXNRD1,GOT2,FN1,CDC42BPA,RSL1D1,RPS28,RPS20,CALM1
**ASH2L**	1.7E-05	3.9E-04	RPL5,COX7B,HIBADH,MRPS14,NDUFA12,PTEN,LAMC1,COX6A1,RPL8,MRPL35,RPL9,PITPNC1,RPL6,RPS15,RPS4X,UBL5,RPS14,SMPD2,RPS16,CSRP2,RPS18,RPL36,IDH3B,

			UQCRFS1,RPL38,DLAT,RPS10,LMCD1,RPS13,FNBP1,RPS5,ITPK1,TALDO1,SDHC,RPS3A,SDHD,PGM2L1,DNAJC3,EEF1A1,HADHA,PLCB4,NDUFS4,RPL24,SPARCL1,SUCLG1,GAS6,PRPS1,OAT,NDUFB7,UQCRB,NDUFB6,ATP5A1,COX7A2,PDHB,ATP5H,ATP5O,UQCRH,ADH5,ATP5L,RPS15A,ALDH1B1,PDGFC,ABL1,COX10,ATP6V1F,ATP6V0A1,NDUFA9,BCHE,DARS,RPL41,MDH1,NDUFA4,IDH2,NDUFA2,H3F3A,RPL35A,COX6C,MRPL24,SOD1,RPS26,RPS25,RSL1D1,GSTA4,MSRB2,CALU,RPS20, CHPT1,SERINC1,NDUFAF1,CALM1,CALM2,ITM2B,FARSB
**EST1**	2.1E-05	4.7E-04	NDUFB5,NDUFB3,ITPR1,NEDD8,MTR,ATP5O,COX7C,RPL9,UQCRH,RPL6,MRPL11,ATP5L,MRPL42,RPS14,RPS18,IDH3B,COX10,RPL41,RPS9,MDH1,RPS5,ECH1,MRPS21,RPL35A,COX6C,MRPL24,PGM2L1,RPIA,RPS26,RPS25,SPCS2,RPS27,RPS29,NDUFS5, YME1L1,RPL27,SERINC1,UQCRC2
**NR0B1**	2.6E-05	5.3E-04	NDUFA12,ATP5C1,PYGM,SLC2A3,ATP5G3,CYR61,RPS14,IMPA2,RPL36,ACADM,RPS10,PGAM1,ITPK1,SARS,SDHC,SDHD,HADHB,HADHA,SMS,SLC25A5,GAPDH,PRPS1,OAT,NDUFB6,MGST3,ATP5A1,GATA6,ATP5J,PDHB,PPM1K,COX5B,ATP5O,COX5A,ATP5L,CST3,ATP5B,ALDH1B1,ATP5E,PDGFC,CKB,NDUFV2,RPL17,COX10, RPL19,GOT2,FN1,COX6C,ASS1,RPS26,GSTA4,CALU,OGDH,MSRB2,ACO1,CHPT1
**ELK1**	4.0E-05	8.0E-04	RPL30,MRPS14,NDUFB3,MGST1,COX7A2,UQCRH,CYR61,GLS,RPS14,CSRP2,RPS19,PPP2R1A,RPS18,RPL38,PRKACB,RPL19,COX8A,PDHX,DARS,RPL41,NDUFA4,ECH1,SIAH1,MRPS21,RPL35A,HADHB,DNAJC3,RPS27,NDUFS5, RPL24,MSRB3,PPARG, SUCLG1,RPS20,NDUFAF1
**CTCF**	5.3E-05	9.9E-04	RPL5,APP,HIBADH,MRPS14,SLC2A1,SLC2A3,SLC2A4,ATP5G2,RPS15,RPS14,RPS19,RPL18A,RARS,RPL36,PMPCB, CYP1B1,RPL35,RPL38,RPL37,FNBP1,RPS5,ITPK1,RPL13A, MRPS18A,RPS3A,ATP5F1,RPL27,RPL26,NDUFB7,UQCRB, NDUFB10,PDE1C,COX15,MGST3,GATA6,COX5A,LTBP1, UQCRH,MRPL11,PPP2CB,OXCT1,ATP5D,NDUFV2, NDUFA6,USP9X,GOT2,ASS1,RPS26,RPS29,NDUFAB1,RPS20
**SOX17**	5.1E-05	9.9E-04	APP,COX7B,MRPS14,COX4I1,SLC2A1,LAMC1,SLC2A4,PIK3C2A,ATP5G2,MRPL35,CYR61,HK2,UBL5,RPS16,RARS, PMPCB,RPL38,ACADM,RPL37,RPS11,COX8A,GSTO1,GPX3,RPS5,APLP2,ECH1,RPL13A,PLA2G4A,SDHC,MRPS18C,EEF1A1,DPYD,NDUFS4,PRPS1,PPM1L,ODC1,GATA6,ITPR3,COX7A2,PDHB,PPM1K,ATP5O,UQCRH,MRPL11,CST3,UBB,RPS3,ATP6V1F,RPL41,NDUFA6,USP9X,IGF1,SOD1,RPS26,RPS25,RPS27, GSTA4,MSRB3,RPS20,CALM1
**FOXO3**	5.9E-05	1.1E-03	PIGT,YWHAB,ATP5G3,LAMC1,PIK3C2A,RPL9,RPL6,RPL18A,AUH,ALDH2,UQCRFS1,RPL37,COA5,RPS9,GSTO2,GSTO1,MRPS18A,SDHC,SDHD,EEF1A1,YME1L1,UQCRC2, RPL26,OAT,NDUFB7,NDUFB6,MRPS36,COX15,NDUFB5, COX5B,ADH5,MRPL11,ACAT1,ATP5L,RPL14,TMEM208, RPL15,RPS2,NDUFV2,COX10,ATP6V1F,NDUFA9,USP25,

			NDUFA6,MDH2,NDUFA4,IDH2,RPL35A,GATM,SPCS2,RPS27,GNPDA2,CYCS, ACO1,CALM3,SERINC1,RPS21,CALM2,ITM2B,FARSB,RPS23
**TAF7L**	8.4E-05	1.5E-03	ACAA2,NDUFB10,COX15,PTEN,MGST1,ATP5J,PDHB,ATP5G3,COX5A,CST3,SMPD2,LGALS1,RPS19,UBB,PDK4,RPL38,ACADM,DLAT,LMCD1,AOC3,GSTO1,NDUFA2,ECH1,RPS3A,MRPS7,ALDH6A1,SDPR,CAT,CALU,CYCS,RPL27,CALM1,CALM2, ALDH9A1
**CREB1**	9.6E-05	1.7E-03	PRPS1,RPL5,UQCRB,NDUFB6,MRPS14,RPL12,AK2,MTR,PWP1,ATP5B,RPS14,RPS19,RPL18A,RPS18,RPL37,RPL18,AASS,RPS13,NDUFA6,PDHA1,MDH1,RPS5,MRPS21,SDHC,MRPL24,MUT,MRPS18C,RPS28,RPS29,CALU,OGDH,RPS20,DLD,CALM2,RPS21
**HOXB4**	1.0E-04	1.7E-03	COX7B,RPLP1,AMD1,PTEN,COX6A1,PITPNC1,UBE2L3,UBL5,MRPL3,PCMT1,RPS19,RPL18A,PPP2R1A,RPS18,RPL38,PRKACB,ECH1,MRPS21,RPL13A,PLA2G4A,ACSL3,EEF1G,HADHB,NDUFS3,RPL24,SUCLG1,MFGE8,NDUFB9,UQCRB,PPM1L,NDUFB3,COX7A2,PDHB,PPM1K,COX5B,OAZ1,COX7A1,UQCRH,ATP5L,PPP2CA,ATP5B,UBB,EIF4EBP2,RPL13,ATP6V0A1,NDUFA9,NDUFA4,GOT2,PHKB,MRPL24,RPS26,RPIA, CALU,MSRB2,RPS21,PICALM
**JARID1A**	1.0E-04	1.7E-03	RPL4,PIGT,COX4I1,RPLP0,ATP5C1,MRPL35,RPL9,PWP1,UBL5,MRPL3,RPS16,RPL18A,AUH,RPS18,IDH3B,UQCRFS1,DLAT,PRKACB,COX8A,RPS5,MRPS21,MRPS18A,SDHC,SDHD,COX6B1,MRPS18C,NDUFS4,NDUFS3,YME1L1,RPL27,DLD,NDUFB9,NDUFB10,NDUFB6,COX15,NDUFB5,MGST3,NDUFB3,NEDD8,COX7A2,ATP5H,COX5B,ATP5D,RPL14,TMEM208,RPS2,RPL18,COX10,ATP6V0A1,RPL19,NDUFA9,LAMB2,NDUFA2,NDUFA1,COX6C,RSL1D1,ALDH6A1,RPS28,SPCS2,NDUFAF2,SERINC1, NDUFAF1,RPS21,FARSB
**FOXP3**	1.0E-04	1.7E-03	NDUFB6,MRPS36,RPL12,MGST3,RPLP0,NDUFB3,PTEN,ATP5J,MIA3,SLC2A3,ATP5G3,UQCRH,MRPL11,MRPL3,RPS14,RPS15A,RPS19,UBB,RARS,TMEM208,RPL38,RPL15,RPL37,RPS2,RPL18,NDUFA9,MDH1,RPL21,NDUFA4,ECH1,COX6C,MRPL24,MRPS18C,EEF1A1,RPIA,ITPKB,RPS26,NDUFS4,NDUFS3,RPL24,SUCLG1,RPS20,UQCRC2, NDUFAF1,RPL26,FARSB
**KLF4**	1.4E-04	2.1E-03	RPL4,RPL5,COX7B,NDUFB7,RPL12,GATA6,PTEN,ITPR3,SLC2A3,RPL8,ADH5,RPS15A,RPL18A,UBB,PPP2R1A,RPS18,ATP5E,PDGFC,ABL1,RPL38,ACADM,RPL15,RPL37,RPS11,RPL17,RPL19,RPL41,RPS9,RPL21,IGFBP5,RPS5,ECH1,ITPK1,FN1,RPL13A,MUT,ITPKB,RPS25,SPCS2,PDE10A,NDUFS4,SMS,RPL27,RPS20,GAPDH,UBA52,PICALM, RPS23
**GATA4**	1.8E-04	2.7E-03	ACAA2,MRPS14,NDUFA12,COX4I1,COX6A1,MRPL35,PRSS23,PITPNC1,HK2,RPS14,CSRP2,PDK4,PELO,IGFBP5,GPX4,FNBP1,RPS5,ITPK1,RPL13A,PGM2L1,COX6B1,HADHB,HADHA,PLCB4,SDPR,PLSCR4,UQCRC2,GAS6,ALDH9A1,OAT,ODC1,GMPS,PPM1K,COX7A1,LTBP1,UQCRH,ADH5,ACAT1,CST3,ATP5B,UBB,ATP6V0A1,ATP6V1F,NDUFA9,SLC35A1,DARS,H3F3A,IGF1,ASS1,SOD1,RPIA,SPC

			S3,RSL1D1,CYCS, CALM3,CALM1,PAM,CALM2,FARSB,PICALM
**DCP1A**	1.9E-04	2.7E-03	UQCRB,RPLP0,SLC2A1,RPL8,OAZ1,ATP5L,RPS16,LGALS1,CYP4V2,RPL37,RPL21,MDH1,NDUFA4,PGAM1,GOT2,RPL13A,RPL35A,MRPS7,MRPL24,RPS26,NDUFS3, CYCS,CALM3,RPS20,CALM1,MFGE8,CALM2,UBE2M,PICALM
**ZFP42**	1.9E-04	2.7E-03	YWHAE,RPL4,RPL5,PIGT,UQCRB,NDUFB5,MAOA,RPL12,UQCRH,ATP5L,UBE2L3,ATP5B,RPS14,UBB,ALDH2,PPP2R1A,RPS18,RPL36,ACADM,RPL15,CKB,RPS11,PELO,NDUFA9,PDHX,RPS9,NDUFA6,GSTO2,PGAM1,NDUFA2,ECH1,MRPS21,RPL13A,RPL35A,MRPS18A,PHKB,MRPS7,MRPL24,ASS1,EEF1A1,HADHB,HADHA,GSTA4,RPL24,YME1L1,ITM2B,RPS23
**HCFC1**	6.0E-04	7.4E-03	MRPS14,MDH2,NDUFB5,ATP5F1,UQCRH,MRPL11,RPIA, RPS28,RPS19,RPS18,COX11,RPL27,SUCLG1,RPL26,RPS23
**ESR1**	6.4E-04	7.8E-03	PRKAA2,PDHA1,ACAA2,NDUFA4,GOT2,GATA6,MGST1,ASS1,ATP5L,SOD1,C3, PCMT1,UBB,ALDH2,PDK4,RPL15,B2M,PELO,PICALM
**PPARD**	9.9E-04	1.1E-02	ITPKB,HADHB,HADHA,CSRP2,OXCT1,ITPR1,PLA2G4A, PRKCA,SDHD,MFGE8, ALDH9A1
**RARG**	1.0E-03	1.1E-02	PIGT,PPM1L,NDUFB10,NDUFA4,MMP2,FN1,RPL13A,LAMC1,FSTL1,LTBP1,PIGY, PITPNC1,ESD,IDH3B,CALU,ACADM,RPL19
**CEBPD**	1.0E-03	1.2E-02	CHRM2,HIBADH,RPLP0,PTEN,PRSS23,CYR61,HK2,RPS14,LGALS1,AUH,PDK4,CYP1B1,RPS13,RPS9,GSTO2,GSTO1,MMP2,MRPS18A,RPS3A,NME4,PLCB4,CAT,NDUFS4,PPARG,GAS6,SLC25A5,UBA52,MGST3,ITPR1,MGST1,ATP5O,FSTL1,LTBP1,ATP5L,UGP2,UBB,PDGFC,ABL1,COX10, ATP6V0A1,ATP6V1F,USP25,FN1,COX6C,ASS1,RPIA, CALU,CALM2,ITM2B,FBN1
**NFE2L2**	1.2E-03	1.2E-02	RPL5,PYGB,CHRM3,OAT,MAOA,NDUFB3,TNC,LAMC1, ATP5G3,ACACB,RPS15A, PDGFC,ESD,CYP1B1,IGFBP7,S1PR3,LMCD1,FNBP1,TXNRD1,FN1,PLA2G4A,TALDO1,MRPS18A,IGF1,COX6C,CDC42BPA,SPCS3,PIK3CA,GSTA4,SDPR,DPYD,CAT,CHPT1,PAM
**NRF2**	1.2E-03	1.2E-02	RPL5,PYGB,CHRM3,OAT,MAOA,NDUFB3,TNC,LAMC1, ATP5G3,ACACB,RPS15A,PDGFC,ESD,CYP1B1,IGFBP7,S1PR3,LMCD1,FNBP1,TXNRD1,FN1,PLA2G4A,TALDO1,MRPS18A,IGF1,COX6C,CDC42BPA,SPCS3,PIK3CA,GSTA4,SDPR,DPYD,CAT,CHPT1,PAM
**YAP1**	1.2E-03	1.2E-02	YWHAE,DGKG,CHRM3,APP,TNC,SLC2A1,SLC2A3,ATP5G3,RPL8,COX7C,CHRDL1,PITPNC1,RPL6,GLS,PCMT1,AUH,PDK4,PGM5,RPL38,RPL37,RPS9,GLRX3,PRKCB,RPS5, APLP2,RPL13A,PLA2G4A,PRKCA,EEF1A1,ITPKB,INPP4B,DPYD,NDUFS4,SPARCL1,PPARG,SUCLG1,DLD,PPM1L, PDE1C,COX5A,ACACB,PPP2CA,ATP5B,UGP2,UBB,RPS3, IGFBP7,S1PR3,COX10,LAMB2,IDH2,GOT2,FN1,PHKB,COX6C,SOD1,SPCS3, PDE10A,GNPDA2,NDUFAB1,OGDH,ACO1,CALM2
**TCFAP2C**	1.3E-03	1.4E-02	PYGB,PIGT,YWHAB,RPLP1,COX4I1,AMD1,SLC2A1,LAM

			C1,COX6A1,PIK3C2A,RPL8,PRSS23,PITPNC1,CYR61,HK2,RPL6,UBL5,PCMT1,LGALS1,RPS18,CYP1B1,RPL35,RPL38,ACADM,RPS11,LMCD1,IGFBP5,GPX4,FNBP1,APLP2,ECH1,ITPK1,SDHC,RPS3A,SDHD,EEF1G,HADHB,UGDH,MFGE8,PRPS1,OAT,PPM1L,ATP5A1,ODC1,GATA6,ITPR1,NEDD8,ITPR3,COX7A2,COX5A,ATP5L,PPP2CA,UGP2,UBB,OXCT1,ATP6V0A1,ATP6V1F,LAMB2,TXNRD1,IDH2,H3F3A,FN1,SOD1,RPIA,CALU,MSRB3,CALM1,PAM,RPS21,PICALM
**NANOG**	2.0E-03	2.0E-02	PRPS1,RPL30,YWHAB,SLC2A3,ATP5G2,RPL9,CYR61,HK2,MRPL11,CST3,CSRP2,RPS19,UBB,ALDH1B1,PMPCB,PIP4K2A,RPL15,RPL18,NDUFA9,PDHA1,APLP2,IDH2,PHKB,IGF1,GSTA4,SMS,CALM3,RPS23
**CUX1**	2.2E-03	2.2E-02	CHRM2,AMD1,SLC2A1,MTR,SLC2A3,ATP5G3,COX7C,HK2,RPS4X,C4A,UBL5,MRPL42,PCMT1,RPS16,RPS19,RPS18,RARS,PMPCB,RPL37,DLAT,RPS11,B2M,RPS13,PDHX,RPL21,MRPS21,SARS,MRPS18A,SDHC,SDHD,MUT,EEF1A1,HADHB,DNAJC3,HADHA,SDPR,PPARG,SUCLG1,RPL27,UQCRC2,PRPS1,NDUFB9,FH,OAT,UQCRB,PPM1L,MGST3,GATA6,GSTT1,TXN,LTBP1,UQCRH,MRPL11,PPP2CA,ATP5B,ESD,RPL14,RPL15,RPL18,RPL17,RPL19,ATP6V1F,USP25,GSN,MDH1,NDUFA2,H3F3A,COX6C,SOD1,RPIA,RPS28,PDE10A,GSTA4,RPS29,PAM,CALM2,UBE2M
**ESR2**	2.4E-03	2.3E-02	PRPS1,ATP6V0E1,WFS1,MAOA,SLC2A1,PLOD2,LAMC1,PITPNC1,BACE1,UGDH, PLA2G16,RPL13,PIP4K2A,CYP1B1,S1PR3,CHPT1,LMCD1
**CCND1**	2.8E-03	2.4E-02	YWHAE,RPL4,APP,PIGT,MRPS14,RPLP1,PTEN,LAMC1,SLC2A4,ATP5G2,PITPNC1, CYR61,UBL5,PCMT1,RPS16,RPS19,ALDH2,PPP2R1A,RPL36,PMPCB,ATP6V1E1,PELO,PLA2G12A,RPS9,IGFBP5,RPS5,TALDO1,SARS,MUT,PGM2L1,HADHB,HADHA,OAT,NDUFB10,NDUFB6,MGST3,AK2,ITPR1,PPM1K,ATP5O,OAZ1,ATP5L,ATP5B,UBB, ATP5D,RPL18,RPL19,ATP6V1F,WFS1,GOT2,NDUFA2,FN1,RPS25,ALDH6A1,OGDH,MSRB2,SERINC1
**OLIG2**	2.7E-03	2.4E-02	CHRM3,APP,PIGT,PTEN,PLOD2,MTR,PRSS23,PLB1,AUH,RARS,PDK4,SLC16A7, RPL38,DLAT,PRKACB,LMCD1,COA5,PDHX,ATP6V0E1,GLRX3,SARS,PRKCA,HADHA,INPP4B,PLCB4,SDPR,NDUFS5,PLSCR4,DPYD,NDUFS4,PRPS1,PDE1C,MAOB,MAOA,GATA6,ITPR3,OXCT1,IGFBP7,DARS,USP25,NDUFA4,PLCL1,PHKB, CDC42BPA,ASS1,SOD1,RPIA,GATM,RPS27,GNPDA2,RPS29,CALU,MSRB2,PAM, PLCD4
**FOXM1**	2.8E-03	2.4E-02	HIBADH,COX4I1,RPLP0,TNC,SLC2A1,PLOD2,ATP5G3,LAMC1,ATP5G2,PITPNC1,CYR61,GLS,MRPL42,ATIC,SLC16A7,PIP4K2A,CYP1B1,ACADM,COX8A,PDHX,IGFBP5,SLC2A12,PLA2G4A,ACSL3,PGM2L1,CIT,EEF1G,UGDH,INPP4B,PLCB4,SDPR,NDUFS4,SMS,SPARCL1,PPARG,SUCLG1,NDUFB9,PPM1L,PDE1C,ODC1,ITPR1,MGST1,PPM1K,COX5B,LTBP1,PPP2CA,RPS15A,UGP2,BCHE,MDH1,PLCL1,TXNRD1,CDC42BPA,CALM2

**TBX20**	2.8E-03	2.4E-02	YWHAE,DGKG,PYGB,RPLP0,SLC2A1,MTR,ATP5G2,RPL8,RPL9,CYR61,HK2,ATIC,IMPA2,PDK4,LMCD1,GSTO2,GPX3,PRKCA,CIT,EEF1A1,DNAJC3,PLCB4,PIK3CA,PPARG,RPL27,GAS6,SLC25A5,PRKAA2,NDUFB7,PDE1C,SDC2, MGST3,ATP5A1,GATA6,ITPR1,PDHB,ATP5H,COX5B,ACACB,ACAT1,CST3,RPS15A,UGP2,ABL1,EIF4EBP2, IGFBP7,KDSR,IP6K2,GSN,MDH1,IDH2,GOT2,FN1,FBN1
**FOXA1**	2.8E-03	2.4E-02	PYGB,RPL5,MRPS14,RPLP1,SPPL2A,LAMC1,PRSS23,CYR61,MRPL42,ATIC,ACADM,LMCD1,COX8A,GLRX3,GPX4, GSTO1,PGAM1,APLP2,TALDO1,MRPS21,ATP5F1,PGM2L1,UGDH,PLA2G16,PPA1,NDUFS5,DPYD,RPL26,GAPDH, ALDH9A1,PRKAA2,ADH1C,MGST3,AK2,UQCR11,PPM1K,UQCRH,ATP5L,UGP2,COX11,KDSR,IGFBP6,ATP6V0A1,CYP2J2,SLC35A1,RPL41,H3F3A,CDC42BPA,RPS26,CYP2C9,RPS27,NDUFAF1, CALM2,PICALM
**ERG**	3.3E-03	2.8E-02	YWHAE,RPL4,RPL5,PIGT,NDUFA11,RPLP0,RPS15,ATIC, RPS16,IMPA2,IDH3B,PMPCB,IDH3A,RPL21,GPX4,GSTO1,RPS5,TALDO1,PGM2L1,EEF1G,PLSCR4,RPL24,YME1L1,SUCLG1,RPL26,ALDH9A1,PRPS1,ATP5A1,ODC1,GMPS,AK2,ITPR1,ITPR3,COX5B,UBB,OXCT1,ATP5D,RPL14,COX11,TMEM208,RPL13,RPL17,ATP6V1F,MDH2, TXNRD1,RPL35A,SOD1,CALU,OGDH,MSRB2,ACO1,RPS21,RPS23
**MYCN**	3.8E-03	3.2E-02	YWHAE,ACAA2,HIBADH,PIGP,LAMC1,RPL9,PITPNC1, GLS,RPS4X,PCMT1,RPS16, RPL38,RPS11,PGAM1,RPL13A,MRPS18A,PGM2L1,PPA1, UBA52,PPM1L,COX15, NDUFB5,MAOA,ODC1,ITPR1,ITPR3,PDHB,COX5A,UQCRH,ADH5,ATP5L,PPP2CB, UGP2,UBB,ATP5D,RPL14,RPL19,RPL41,WFS1,TXNRD1,H3F3A,FN1,RPL35A,PHKB,ASS1,SOD1,RPS26,ALDH6A1,RPS28,RPS29,NDUFAB1,RPS20,CALM3,NDUFAF1, CALM1,PAM,RPS21,CALM2,PICALM
**PPAR**	4.1E-03	3.4E-02	PYGB,FH,MAOA,MGST3,GATA6,AK2,PLOD2,ATP5O,FSTL1,LTBP1,HK2,MRPL3, AUH,PDGFC,RPS3,PDK4,CYP1B1,BCHE,TXNRD1,ECH1, SIAH1,SARS,MRPS18A, ACSL3,UQCRHL,EEF1A1,HADHB,HADHA,INPP4B,NDUFS5,CAT,ACO1,RPL26, PICALM,FBN1
**SALL4**	4.9E-03	4.0E-02	NDUFB6,NDUFB5,MRPS14,MGST3,AMD1,PTEN,ATP5I,SLC2A3,ATP5G3,PRSS23, PCMT1,RPS15A,RPS16,UBB,ABL1,IDH3B,IGFBP7,IGFBP6, NDUFV2,RPS11,PELO,B2M, SLC35A1,ENTPD1,RPL41,RPS9,MDH1,NDUFA4,MMP2,RPS5,H3F3A,SARS,PLA2G4A, HADHA,PLSCR4,CYCS,RPL27,RPS20,RPL26,RPS23
**EGR1**	5.4E-03	4.3E-02	HADHB,NDUFB9,H3F3A,ITPK1,GMPS,PIP4K2A,ATP5I,KDSR,ATP5H,CYP4F11, CRYM,PRSS23
**FOXP2**	5.5E-03	4.3E-02	APP,HIBADH,YWHAB,PIGP,ADARB1,PLB1,PCMT1,UGP2,AUH,UBB,RARS,PDGFC,PDK4,EIF4EBP2,PIP4K2A,NDUFV2,USP25,ATP6V0E1,GOT2,H3F3A,PLA2G4A,MUT, RPIA,ITPKB,SPCS2,GSTA4,CYCS

**THAP11**	5.5E-03	4.4E-02	YWHAE,NDUFB7,ITPR1,ATP5J,NEDD8,MTR,ATP5O,ATP5G2,COX7C,RPS18,ATP5E,RPL36,RPS3,RPL15,RPL18,ATP6V1F,MDH1,GOT2,PGM2L1,RPIA,BACE1,RPS26, SPCS2,SUCLG1,RPS20,RPL26,RPS23
**FUS**	6.1E-03	4.7E-02	PRKAA2,MAOA,PLA2G4A,PHKB,MTR,ATP5G3,RPL9,FSTL1,COX7C,MUT,CYR61, ACAT1,EEF1A1,RPS4X,RARS,ATP5E,RPL15,PAM,GAPDH

NOTE: FC= Fold Change; FDR p-val= FDR p-value.

Table S6. Putative binding motif enrichment analysis using the Enrich webtool and showing the relative relevance of various histone marks in the deregulated transcripts.

Term	p-value	Adj. *p*-val	Genes
**H3K79me2**	7.2E-24	3.0E-21	NDUFA11;NDUFA12;ATP5C1;COX6A1;RPL9;RPL6;RPS15;LGALS1;RPS16;RPL18A;RPS18;RPL35;PMPCB;RPL37;RPS11;RPS10;RPS13;PDHX;RPS9;IGFBP5;RPL21;SDHC;SDHD;MRPS18C;COX6B1;EEF1A1;RPL27;RPL26;UBA52;UQCRB;SDC2;ITPR3;ATP5O;OAZ1;UQCRH;ADH5;ATP5B;UBB;ESD;NDUFV2;COX10;ATP6V0A1; DARS;RPL41;ATP8B2;MRPL24;RPS26;RPS25;RPS28;NDUFAF4;CALU;CYCS;RPS20;ACO1;RPS21;RSL24D1;RPS23;COX20;MRPS14;RPLP1;RPLP0;ATP5G3;PFAS;CYR61;RPS4X;UBL5;MRPL3;CSRP2;ATIC;IDH3B;COA5;GPX4;GSTO1;MRPS21;RPS3A;ATP5F1;MRPS7;CIT;EEF1G;NDUFS5;NDUFS4;DLD;NDUFB7;NDUFB6;RPL12;ATP5A1;NDUFB3;UQCR10;PDHB;COX5B;FSTL1;PPP2CB;RPS3;TMEM208;RPL15;RPS2;RPL18;RPL17;USP25;NDUFA6;LAMB2;NDUFA2;RPL35A;RSL1D1;NDUFAB1
**H3K36me3**	6.4E-18	6.5E-16	COX7B;RPL30;MRPS14;YWHAB;NDUFA12;RPLP1;COX4I1;ECI2;SPPL2A;RPLP0;ATP5G3;COX7C;CYR61;RPS15;MRPL3;RPS14;ATIC;RPS16;ALDH2;RPS19;RPS18;RPL36;RPL35;RPL38;ACADM;RPL37;DLAT;RPS11;PELO;RPS10;RPS13;COA5;COX8A;RPL21;GSTO2;GPX4;ATP6AP1;ECH1;MRPS21;SDHD;ATP5F1;MRPS18C;EEF1A1;EEF1G;PIK3CA;RPL24;RPL27;RPL26;UQCRC2;SLC25A5;GAPDH;UBA52;NDUFB10;COX15;RPL12;ATP5A1;MGST1;ITPR1;ATP5J;PDHB;ATP5H;ATP5O;COX5B;ACACB;ADH5;C3;ATP5B;ATP5E;ATP5D;RPL14;EIF4EBP2;RPL13;TMEM208;RPL15;RPL18;RPL17;RPL19;FN1;IGF1;COX6C;SOD1;RPS26;SPCS3;RPS25;RPS28;RPS27; RPS29;NDUFAB1;CYCS;CHPT1;RPS20;RSL24D1;COX20;RPS23
**H3K79me3**	6.4E-18	6.5E-16	MRPS14;NDUFA12;RPLP1;COX4I1;ATP5C1;PLOD2;COX6A1;COX7C;RPL9;RPL6;RPS4X;RPS15;MRPL42;PCMT1;LGALS1;RPS16;RPS19;RPL18A;RPS18;RPL36;PMPCB;ATP6V1E1;RPS11;RPS10;RPS13;RPS9;GPX4;GLRX3;RPS5;RPS3A;SDHC;SDHD;MRPS7;CIT;COX6B1;EEF1G;EEF1A1;PPA1;NDUFS5;NDUFS4;NDUFS3;RPL27;RPL26;SLC25A5;DLD;UBA52;GAPDH;OAT;NDUFB6;UQCRB;NDUFB5;NDUFB3;COX7A2;COX5B;ATP5O;OAZ1;ADH5;UBB;ATP5E;ESD;RPS3;TMEM208;IGFBP7;RPL15;RPS2;NDUFV2;RPL18;RPL17;RPL19;NDUFA9;RPL41;NDUFA6;MDH1;LAMB2;NDUFA2;H3F3A;RPL35A;RPS25;RSL1D1;RPS28;SPCS2;RPS29;NDUFAF4;NDUFAB1;NDUFAF2;CALU;CYCS;RPS20;CALM3;NDUFAF1;CALM1;RSL24D1;RPS21;RPS23

**H4K20me1**	4.0E-09	7.5E-08	RPL4;RPL5;ACAA2;RPLP1;ECI2;RPLP0;ATP5C1;SLC2A3;ADARB1;ATP5G3;RPL8;CYR61;UBE2L3;RPS15;RPS14;RPL18A;RPL36;RPL35;SLC16A7;RPL38;RPL37;RPS11;RPS9;RPL21;FNBP1;RPS5;MMP2;RPL13A;RPS3A;PRKCA;MRPS7;EEF1A1;UGDH;PPA1;YME1L1;RPL24;SUCLG1;RPL27;UQCRC2;RPL26;UBA52;GAPDH;OAT;MAOB;PPM1L;RPL12;ATP5J;UQCR10;PPM1K;OAZ1;ADH5;ITPRIPL2;ATP5B;UGP2;UBB;ABL1;RPL13;RPS2;RPL15;NDUFV2;RPL18;RPL17;ATP6V0A1;IP6K2;RPL19; MDH2;GOT2;RPS26;RPS28;MSRB3;RPS20;CALM3;ACO1;FBN1
**H3K27ac**	3.0E-07	4.0E-06	CHRM2;ACAA2;NDUFA11;NDUFA12;SPPL2A;COX6A1;PWP1;PCMT1;RPS19;RPL36;PDK4;ATP6V1E1;PELO;PRKACB;RPS10;RPS9;RPL21;IGFBP5;RPS5;MRPS18A;SDHC;ACSL3;SUCLG1;UQCRC2;DGKH;COX15;ITPR1;GSTT1;ITPR3;COX7A2;UQCRH;ITPRIPL2;UBB;ATP5E;ESD;COX11;IGFBP7;COX10;SIAH1;PHKB;RPS26;RPS25;RPS27;RPS29;NDUFAF4;OGDH;SERINC1;NDUFAF1;CALM1;PAM;FBN1;RPS23;FARSB;PYGB;YWHAB;RPLP1;AMD1;SLC2A1;MIA3;MRPL35;PITPNC1;SMPD2;IDH3B;PGM5;AASS;IDH3A;PLA2G12A;GSTO2;SLC2A12;PLA2G4A;MRPS21;MRPS7;CIT;HADHB;HADHA;UGDH;CAT;NDUFS3;NDUFB9;NDUFB5;NDUFB3;ODC1;UQCR10;COX5B;PPP2CB;RPS15A;OXCT1;TMEM208;RPL13;RPL15;RPS2;RPL19;NDUFA9;BCHE;NDUFA6;MDH1;USP9X;NDUFA4;TXNRD1;NDUFA2;RPL35A;RPIA;RSL1D1;SPCS2;MSRB3;PLCD4
**H2AFZ**	1.1E-04	9.8E-04	YWHAE;DGKG;PIGT;HIBADH;NDUFA12;MRPS14;PIGP;SLC2A1;MIA3;ATP5G3;ATP5G2;RPL6;RPS14;CSRP2;ATIC;ALDH2;PPP2R1A;CYP4V2;RPL36;PMPCB;UQCRFS1;PGM5;RPL37;PELO;RPS10;COX8A;PLA2G12A;GPX3;SDHC;ACSL3;MRPS7;ATP5F1;CIT;INPP4B;UGDH;PPA1;SMS;SUCLG1;UBA52;PRPS1;NDUFB9;NDUFB7;NDUFB5;NDUFB3;AK2;COX7A2;PDHB;ATP5L;RPS15A;ATP5D;RPL14;EIF4EBP2;COX10;MDH2;PLCL1;NDUFA2;RSL1D1;RPS20;SERINC1;RSL24D1
**H3K4me3**	6.1E-04	4.3E-03	CHRM2;RPL4;PYGB;SPPL2A;ECI2;SAT2;ATP5G2;CHRDL1;PCMT1;MRPL3;MRPL42;ATIC;ALDH2;PDK4;UQCRFS1;ACADM;DLAT;ATP6V1E1;B2M;AASS;IDH3A;RPS9;GSTO2;ITPK1;ECH1;MRPS7;DPYD;NDUFS4;MFGE8;GAPDH;DLD;PDE1C;NDUFB6;MAOB;NDUFB10;COX15;SDC2;MGST3;ITPR1;FSTL1;ESD;TMEM208;IP6K2;PDHA1;NDUFA4;FN1;NDUFA1;RPS26;RSL1D1;ALDH6A1;RPS27;GSTA4;ACO1;NDUFAF1;PLCD4;FARSB;MSRB1
**H3K9me1**	1.0E-03	6.3E-03	RPL4;RPL30;NDUFA11;RPLP1;RPLP0;ATP5C1;ATP5G3;ATP5G2;GLS;LGALS1;ATIC;RPL36;PMPCB;RPL35;RPL37;RPS11;RPL21;ATP5F1;COX6B1;PPA1;UQCRC2;RPL26;MFGE8;UBA52;SLC25A6;FH;MAOB;COX15;MAOA;RPL12;ACACB;ACAT1;ITPRIPL2;RPS15A;UGP2;ALDH1B1;ATP5E;RPL14;TMEM208;EIF4EBP2;RPL13;RPS2;RPL15;RPL18;RPL17;ATP6V0A1;GSN;ATP8B2;MDH1;MDH2;FN1;UQCRHL; RPS28;GNPDA2;CHPT1;FARSB
**H3ac**	2.8E-03	1.4E-02	NDUFA11;NDUFA12;COX4I1;RPLP1;SLC2A1;ATP5C1;PLOD2;PRSS23;MRPL35;CSRP2;MPC1;IDH3B;ACADM;PRKACB;PELO;RPS10;RPS13;PLA2G12A;GSTO1;APLP2;ITPK1;RPS3A;NME4;SDHD;MRPS7;HADHB;PPA1;NDUFS5;NDUFS3;UQCRC2;PRKAA2;PPM1L;NDUFB5;UQCR11;NEDD8;COX7A2;FSTL1;MRPL11;COX11;TMEM208;NDUFV2;SLC35A1;DARS;USP25;NDUFA6;N

			DUFA4;RSL1D1;NDUFAF4;NDUFAF2;CYCS;MSRB3;ACO1;PICALM;COX20
**H3K9me3**	1.1E-02	4.5E-02	RPL5;PIGT;NDUFA12;YWHAB;RPLP1;RPLP0;ATP5C1;ATP5G2;RPL6;RPS14;RPS16;AUH;RPL18A;ALDH2;RPS19;RPL35;ACADM;RPS10;RPS13;RPL21;RPL13A;RPS3A;SDHD;MRPS7;EEF1A1;HADHB;HADHA;CAT;NDUFS4;RPL27;UQCRC2;UBA52;MAOA;AK2;COX7A2;TXN;ATP5B;RPS3;RPL13;RPS2;RPL18;ATP6V0A1;RPL41; NDUFA6;COX6C;RSL1D1;MSRB2;RPS20;CALM3;RPS21;FBN1

NOTE: FC= Fold Change; FDR p-val= FDR p-value.

Table S7. Assignment of the main metabolites identified in NMR urine spectra.

N. Assigment	Metabolite	Group	Chemical shift (ppm)
2	Branched chain amino acids	--	--
3	Valine	γCH_3	0.98
3	Valine	γCH_3	1.04
4	Methylsuccinic acid	αCH_3	1.08
5	Lactate	CH_3	1.33
6	Alanine	βCH_3	1.46
7	Lysine	βCH_2	1.71
8	Acetic acid	CH_3	1.97
7	Lysine	βCH_2	1.91
9	N-acetylneuraminic acid	CH_3	2.04
10	Glutamine	βCH_2	2.13
11	Succinic acid	2,3 CH_2	2.39
10	Glutamine	γCH_2	2.44
12	Citrate	CH_2	2.52
12	Citrate	CH_2	2.66
13	Dimethylamine	CH_3	2.71
14	Trimethylamine	CH_3	2.93
15	Dimethylglycine	CH_3	2.96
16	Creatinine	CH_3	3.03
19	Taurine	$-CH_2-NH_3^+$	3.25
17	Trimethylamine N-oxide	CH_3	3.28
18	Methanol	CH_3	3.34
19	Taurine	$-CH_2-SO_3^-$	3.42
20	Glycine	αCH	3.55
21	Sucrose	$C_{6'}-H_2$	3.82
21	Sucrose	$C_{5'}-H$	3.87
22	Creatine	CH_2	3.92
23	Hippuric acid	αCH_2	3.97
16	Creatinine	CH_2	4.05
5	Lactate	CH	4.10
24	Trigonelline	CH_3	4.43
25	Urea	$-NH_2$	5.80
26	3-(3-hydroxyphenyl)-3-hydroxypropionic acid (HPHPA)	$C_{4'}-H$	6.93
26	3-(3-hydroxyphenyl)-3-hydroxypropionic acid (HPHPA)	$C_{6'}-H$	6.97

27	Phenylalanine	$C_{2,6'}$-H	7.32
27	Phenylalanine	$C_{3,5'}$-H	7.42
23	Hippuric acid	$C_{3,5'}$-H	7.60
28	Pseudouridine	CH	7.66
23	Hippuric acid	$C_{2,6'}$-H	7.82
29	Hypoxanthine	$C_{7'}$-H	8.20
30	Formic acid	CH	8.45
24	Trigonelline	$C_{3,5'}$-H	8.82
24	Trigonelline	$C_{1'}$-H	9.10

References

1. Martínez-Bisbal, M.C.; Monleon, D.; Assemat, O.; Piotto, M.; Piquer, J.; Llácer, J.L.; Celda, B. Determination of metabolite concentrations in human brain tumour biopsy samples using HR-MAS and ERETIC measurements. *NMR Biomed.* **2009**, *22*, 199–206.
2. Beckonert, O.; Keun, H.C.; Ebbels, T.M.D.; Bundy, J.; Holmes, E.; Lindon, J.C.; Nicholson, J.K. Metabolic profiling, metabolomic and metabonomic procedures for NMR spectroscopy of urine, plasma, serum and tissue extracts. *Nat. Protoc.* **2007**, *2*, 2692–2703.
3. Savorani, F.; Tomasi, G.; Engelsen, S.B. icoshift: A versatile tool for the rapid alignment of 1D NMR spectra. *J. Magn. Reson.* **2010**, *202*, 190–202.
